# Stability of Rayleigh–Jeans Equilibria in the Kinetic FPU Equation

**DOI:** 10.1007/s00205-026-02223-2

**Published:** 2026-07-07

**Authors:** Pierre Germain, Joonhyun La, Angeliki Menegaki

**Affiliations:** 1 Department of Mathematics, Huxley Building, South Kensington Campus, Imperial College London, London, SW7 2AZ UK; 2 June E Huh Center for Mathematical Challenges, 85 Hoegi-ro, Dongdaemun-gu, Seoul, 02455 Republic of Korea

## Abstract

We study the nonlinear dynamics of the kinetic wave equation associated with the FPU problem and prove the stability of the non-singular Rayleigh–Jeans equilibria. The lack of a spectral gap for the linearized problem leads to polynomial decay, which we are able to leverage to obtain nonlinear stability.

## Introduction

### The microscopic model

In their groundbreaking 1955 study [7], Fermi, Pasta, Ulam and Tsingou utilized the early electronic computers to explore the relaxation dynamics (thermalisation) of a chain of coupled nonlinear oscillators. The Fermi-Pasta-Ulam-Tsingou system is an anharmonic chain of oscillators without pinning potential. The system is described by its momentum $(p_n)_{n \in \mathbb {Z}}$ and position $(q_n)_{n \in \mathbb {Z}}$ with the Hamiltonian energy given by

$$\begin{aligned} H(p,q) = \sum _{n \in \mathbb {Z}} \frac{p_n^2}{2} + \sum _{n \in \mathbb {Z}} F(q_{n+1} - q_n), \end{aligned}$$

where *F* is smooth and satisfies F(0)=0.

The dynamics are governed by the system of ODEs

$$ -\ddot{q_n} = F''(q_{n+1}-q_n) - F''(q_n - q_{n-1}). $$

A particular example is the so-called FPUT-β case

$$ F(x) = \frac{1}{2} x^2 + \frac{\beta }{4} x^4, \text {with } \beta >0, $$

which will be the focus of the present article.

Motivated by the original FPUT problem and its substantial impact [4], significant attention has been given to the energy transport within oscillator chains of length *N* coupled to thermal reservoirs at different temperatures. Numerically, it is observed that in β-FPUT chains, the thermal conductivity $\kappa _N$ diverges as *N* increases in an anomalous way, specifically as $\kappa _N \sim N^{2/5}$ [1, 9]. An alternative approach is to consider an infinite chain and observe energy spread after injecting energy at the origin. This reformulates the problem to that of studying the time-decay of the energy current-current correlation function, which is related to the decay rate of the linearized semigroup of the wave turbulence kinetic equation arising from such microscopic models [2]. For the linearised problem of the β-FPUT chain, it was proven in [11] that the correlation decays as $\mathcal {O}(t^{-3/5})$, aligning with numerical findings. Following the study in [11], in [12] it was rigorously derived a macroscopic fractional diffusion equation describing heat transport in β-FPUT chains, confirming the anomalous diffusion behavior from the linearized Boltzmann phonon equation. Our main inspiration and motivation here is also the findings in [11].

We also mention other classical choices of nonlinear atom chains. Beside the β-FPU chain, one may consider the α-FPU chain, where $F(x) = \frac{x^2}{2} + \alpha \frac{x^3}{3}$, or the $\alpha +\beta$-FPU chain, which combines the nonlinearities of both the α and β chains. Moreover, adding an additional pinning potential to the dynamics results in the Discrete Nonlinear Klein-Gordon chain, where $F_{KG}(x) = \frac{\delta }{2}x^2$ and the energy includes $U_{pin}(q) = (\frac{1}{2}-\delta ) q^2 + \frac{1}{4} q^4$. Finally, the Toda lattice is yet another model, where $F_T(x) = \frac{1}{4\alpha ^2}(e^{2\alpha x}-1-2\alpha x)$. For a detailed account and discussion on these models, we refer to the reviews [10, 13, 14].

### The homogeneous kinetic wave equation

In this article, we focus on the kinetic wave equation, or phonon Boltzmann equation, arising from the β-FPUT system. The kinetic wave equation we will consider can be written as

$$ \partial _t f(t,p) = \mathcal {C}[f](t,p), $$

where the collision operator is given by

1.1 $$\begin{aligned} \mathcal {C}[f](t,p_0)=\int _{\mathbb {T}^{3}} \delta (\Sigma ) \delta (\Omega ) \prod _{\ell =0}^3 \omega _\ell \prod _{\ell =0}^3 f_\ell \left( \frac{1}{f}+\frac{1}{f_1}-\frac{1}{f_2}-\frac{1}{f_3}\right) \, \textrm{d}p_1 \, \textrm{d}p_2 \, \textrm{d}p_3, \end{aligned}$$

with the usual notations $p=p_0$, $f= f_0 = f(p_0)$, $f_i=f(p_i)$ if i=1,2,3, and similarly for $\omega _i=\omega (p_i)$, Furthermore,

$$\begin{aligned}&\omega (p) = \left| \sin \left( \frac{p}{2} \right) \right| \\&\Sigma = \Sigma (p, p_1, p_2,p_3) = p_0+p_1-p_2-p_3 \\&\Omega = \Omega (p, p_1, p_2,p_3) = \omega _0 + \omega _1 -\omega _2 - \omega _3. \end{aligned}$$

Finally we denote $\mathbb {T}$ for the periodized torus as

$$ \mathbb {T} = \mathbb {R} / 2\pi \mathbb {Z}. $$

The equation conserves two quantities which will play an important role in our analysis: the mass $\mathcal {M}$ and energy $\mathcal {E}$,

$$\begin{aligned}&\mathcal {M}(f) = \int _{\mathbb {T}} f(p) \, \textrm{d}p \\&\mathcal {E}(f) = \int _{\mathbb {T}} \omega (p) f(p) \, \textrm{d}p. \end{aligned}$$

The equation also satisfies an H-theorem

$$ \frac{d}{dt} \int \log f(p) \, \textrm{d}p \le 0, $$

even though we will not make use of this fact.

Finally, the Rayleigh–Jeans equilibria (RJ)

$$ \mathfrak {f}_{\beta ,\gamma }(p) = \frac{1}{\beta \omega (p) + \gamma }, \qquad \beta ,\gamma \ge 0 $$

are the unique stationary solutions [11, Section 5].

The case γ=0 corresponds to a singular RJ equilibrium, which has infinite mass. However, it is favored by some authors since it gives equipartition of energy, as was initially expected by E. Fermi et.al. in their numerical experiment [10, 13].

Interestingly, not all couples $(\mathcal {M}_0,\mathcal {E}_0)$ can be associated to a RJ equilibrium, if the inverse temperature is positive, i.e. β>0. For initial data whose mass and energy cannot be matched to a RJ equilibrium, the asymptotic behavior of the solution is an intriguing question - see the discussion in Sect. 7 and [6], where we provide a characterization of the entropy maximizers. Allowing also for negative temperatures, we prove that we are always (for any pair $(\mathcal {M}_0,\mathcal {E}_0)$) able to find RJ equilibria having the associated mass and energy.

### Making sense of the collision operator

The expression for the collision operator in (1.1) is not obviously meaningful, even for smooth functions *f*. Indeed, it involves the product of two δ functions, which, as is well-known, can be ill-defined.

As was shown in [11] (see also Lemma 12), the common zeros of Ω and Σ are of two kinds. On the one hand, the trivial zeros are such that $\{ p_0, p_1 \} = \{ p_2, p_3 \}$. The integrand of the collision operator vanishes identically on the set of trivial zeros, which provides some cancellation. However, from the viewpoint of distribution theory, the formal collision kernel involves the singular product $\delta (\Omega ) \delta (\Sigma )$, and so it is not clear how to make sense of the product of two Dirac δ, even if it is evaluated on a function vanishing on their singular set ("indefinite form"). In the linearized setting, Lukkarinen and Spohn in [11] justify the cancellation by by introducing a regularization of the singular resonance constraint and then passing to the limit. In that limit, only the nontrivial branch, cf. (1.2), survives in the final expression for the operator. In that sense, the trivial zeros $\{ p_0, p_1 \} = \{ p_2, p_3 \}$ do not contribute. This regularization is closely related to the microscopic derivation of the equation (which remains an outstanding open problem) and we shall take for granted that trivial zeros can be ignored in the collision operator.

On the other hand, non-trivial zeros can be parameterized by

1.2 $$\begin{aligned} p_1 = h(p_0,p_2) \mod 2\pi , \end{aligned}$$

where

1.3 $$\begin{aligned} h(x,z) = \frac{z-x}{2} + 2 \arcsin \left( \tan \frac{|z-x|}{4} \cos \frac{z+x}{4} \right) . \end{aligned}$$

Using this expression, a calculation ([11] or Lemma 13) shows that the collision operator can be written as

1.4 $$\begin{aligned} \mathcal {C}[f](p_0)= \int _0^{2\pi } \frac{ \omega _0 \omega _1 \omega _2 \omega _3 }{\sqrt{F_+(p_0,p_2)}} \prod _{\ell =0}^3 f_\ell \left( \frac{1}{f}+\frac{1}{f_1}-\frac{1}{f_2}-\frac{1}{f_3}\right) dp_2, \end{aligned}$$

where it is understood that

$$ {\left\{ \begin{array}{ll} &  p_3 = p_0 + p_1 - p_2\\ &  p_1 = h(p_0,p_2). \end{array}\right. } $$

and

1.5 $$\begin{aligned} F_{\pm }(p_0,p_2) = \left[ \cos \left( \frac{p_0}{2}\right) +\cos \left( \frac{p_2}{2}\right) \right] ^2 \pm 4\sin \left( \frac{p_0}{2}\right) \sin \left( \frac{p_2}{2}\right) . \end{aligned}$$

### Main results and organization of the paper

Section 3 establishes basic local well-posedness results in weighted $L^\infty$ spaces. It also shows that local well-posedness cannot be expected (or at least is very delicate) in $L^p$-based spaces for p<3. The results of this section (Theorems 1 and 2) can be summarized as the following theorem:

#### Theorem

The collision operator is bounded in $\omega ^\alpha L^\infty$ for $\alpha > -\frac{5}{4}$. Thus, the equation is locally well-posed in $\mathcal {C} (\omega ^\alpha L^\infty )$ (the space of continuous functions with values in $\omega ^\alpha L^\infty$ for $\alpha > -\frac{5}{4}$).

The collision operator is unbounded on $L^p$ for any p<3.

In Section 4, we turn to the linearized problem around RJ equilibria, recapitulating many results obtained in [11], and providing some extensions.

As explained below, for solutions of the form

$$f_t(p) = \mathfrak {f}_{\beta ,\gamma }(p)(1+g_t(p)),\ \quad \beta ,\gamma >0,$$

that is, perturbations around RJ equilibria $\mathfrak {f}_{\beta ,\gamma }(p)$, the linearized evolution of *g* is governed by the semigroup $e^{tL}$ which is generated by the operator *L* given by

$$[L g](p) = \frac{1}{\mathfrak {f}(p)} \int _{\mathbb {T}^3} \delta (\Sigma ) \delta (\Omega ) \left[ \prod _{\ell =0}^3 \omega _\ell \mathfrak {f}_\ell \right] \left[ -\frac{g}{\mathfrak {f}}-\frac{g_1}{\mathfrak {f}_1}+\frac{g_2}{\mathfrak {f}_2} +\frac{g_3}{\mathfrak {f}_3}\right] \,\, \textrm{d}p_{1} \, \textrm{d}p_{2} \, \textrm{d}p_{3}. $$

This is a symmetric operator on the unweighted $L^2(\mathbb {T}):= L^2(\mathbb {T}, \, \textrm{d}p)$ space, endowed with the usual inner product $\langle f,g \rangle = \int _{\mathbb {T}} f(p) g(p) \, \textrm{d}p$. Also, it can be proven, cf. Lemma 1, that $\operatorname {Ker} L = \text {span}\{\mathfrak {f},\mathfrak {f}\omega \}$. We denote by Π the $L^2(\mathbb {T})$ - orthogonal projection onto $\operatorname {Ker} L$. To be more precise, $\Pi g \in \operatorname {Ker} L$ is the unique element such that

$$(\operatorname {Id} - \Pi ) g \perp \operatorname {Ker} L \ \ \text { in } L^2. $$

The traditional tools of kinetic theory only yield a weighted $L^2_{t,p}$ type estimate for the perturbation (Corollary 1), where the weight degenerates.

#### Theorem

We denote by *L* the linearized operator around a RJ solution with $\beta ,\gamma >0$. There exists a function *a*(*p*) satisfying $|a(p)| \sim |\sin \left( \frac{p}{2} \right) |^{\frac{5}{3}}$ such that for all initial data $g_0 \in L^2(\mathbb {T})$ with

$$ \int _0^{2\pi } a(p) g_0(p) \phi (p) \, \textrm{d}p = 0 \ \text { for all } \phi \in \operatorname {Ker} L,$$

it holds that

$$ \int _0^\infty \int _0^{2\pi } a(p) |e^{tL} g_0(p)|^2 \, \textrm{d}p \, \textrm{d}t \lesssim \Vert g_0 \Vert _{L^2}^2. $$

This estimate is insufficient for nonlinear purposes: the decay in time is weak, and we cannot hope to close nonlinear estimates since the equation is ill-posed in $L^2$ type spaces. Furthermore, the lack of a spectral gap leads to the degenerate weight *a*(*p*).

In Section 5 we address these shortcomings by proving pointwise decay at a polynomial rate (Theorem 3).

#### Theorem

Let *L* be the linearized operator around a RJ solution with $\beta ,\gamma >0$. Assume that $g_0 \in L^\infty$, with a zero projection (in $L^2$) on $\operatorname {Ker} L$, i.e. $\Pi g_0=0$. Then, for any $\mu ,\nu \in [\frac{1}{6}, \frac{1}{2}]$ and any δ>0,

$$ \Vert \omega ^\mu e^{tL} g_0 \Vert _{L^\infty } \lesssim _\delta \langle t \rangle ^{- \frac{3}{5}(\mu + \nu )+ \delta } \Vert \omega ^{-\nu } g_0 \Vert _{L^\infty }. $$

This is achieved by understanding how the edges of the frequency domain, where dissipation degenerates, interact with the bulk of the domain. At a more technical level, we resort to an iterative scheme to gain decay increasingly.

Finally, Section 6 deals with the fully nonlinear problem, showing global existence of solutions around non-singular RJ. This is accomplished through a careful definition of an appropriate norm that allows us to control the nonlinearity, relying crucially on the structure of the equation (Theorem 4).

#### Theorem

There exists $\epsilon _0$ such that the following holds: if

$$ f(t=0) = \mathfrak {f}_{\beta ,\gamma } [ 1 + g_0], $$

where $\int \mathfrak {f}_{\beta ,\gamma } g_0 \, \textrm{d}p = \int \omega \mathfrak {f}_{\beta ,\gamma } g_0 \, \textrm{d}p = 0$ and $\Vert \omega ^{-\frac{1}{2}} g_0 \Vert _{L^\infty } = \epsilon < \epsilon _0$, then there exists a global solution which can be written as

$$ f(t) = \mathfrak {f}_{\beta ,\gamma } [ 1 + g] \qquad \text{ where } \quad \Vert \omega ^{\frac{1}{2}} g(t,p) \Vert _{L^\infty _p} \lesssim \epsilon \langle t \rangle ^{-\frac{3}{5} + \frac{1}{1000}} \quad \text{ for } \text{ all } t \ge 0. $$

The appendix gathers some elementary but involved computations which are used in the rest of the text.

### Stability of equilibria for the Boltzmann equation

It is worthwhile comparing the results which have been stated above to analogous statements for the spatially homogeneous Boltzmann equation. In the case of the classical Boltzmann equation, the nonlinearities are quadratic, unlike the cubic nonlinearities of the wave turbulence equation, and the equilibrium is the Maxwellian distribution. Depending on the collision kernel, it is known that the linearized operator around the equilibrium possesses a spectral gap (for instance in the hard sphere case), leading to exponential convergence to equilibrium. However, in the soft potential case, there is no spectral gap, and the decay of the linearized operator is at most of order $e^{-t^{\sigma }}$, σ<1. For more details, see the reviews and articles [3, 8, 16] and references therein.

Roughly speaking, the slightly weaker decay under soft potentials can be attributed to the fact that the collision frequency in the kernel of the Boltzmann operator is not lower-bounded and degenerates at large velocities. However, since the perturbative regime is around a Maxwellian distribution, the initial data roughly resembles a Maxwellian, making the large velocity regime, where the spectral gap becomes zero, less significant.

In our case, we also encounter a degenerate spectral gap that becomes zero at the edges of the frequency domain. This type of degeneracy is very different in nature and significantly affects the decay of the linearized semigroup.

Finally, the spatially inhomogeneous Boltzmann equation has been extensively studied, presenting additional mathematical challenges related to the existence of local equilibria. The theory of hypocoercivity has been developed for such inhomogeneous kinetic models, see [5, 15, 17] and references therein.

## Notations

For quantities *A* and *B* and parameters *a*, *b*, *c*, we write $A \lesssim _{a,b,c} B$ if there exists a constant *C*(*a*, *b*, *c*) such that A≤C(a,b,c)B.

We write A∼B if A≲B and B≲A.

We write *C* for a positive constant whose value may change from line to line.

If $b \in \mathbb {R}$, an exponent b+ is to be interpreted as follows: $f(t) \lesssim t^{b+}$ means that

$$ f(t) \lesssim _\epsilon t^{b+\epsilon } \qquad \text{ for } \text{ any } \epsilon >0. $$

Finally, we denote by $\langle \cdot , \cdot \rangle$ the usual $L^2$ inner product. The space $L^2_a$ is the $L^2$ space on $\mathbb {T}$ endowed with the measure $a(x) \, \textrm{d}x$, with the inner product

$$ \langle f,g \rangle _a:= \int a(p) f(p) g(p)\, \, \textrm{d}p. $$

The notation $g \perp _{L^2_a} \operatorname {Ker}(L)$ means the function *g* is orthogonal to the subspace $\operatorname {Ker} L$ with respect to the weighted inner product. Sometimes we also write $g \in (\operatorname {Ker} L)^{\perp _a}$, with the index *a* stressing that the orthogonality is wrt the weighted geometry.

## Local well-posedness

### Local well-posedness in weighted $L^\infty$ spaces

We consider here the Cauchy problem

$$ {\left\{ \begin{array}{ll} &  \partial _t f = \mathcal {C}[f](p)\\ &  f(t=0) = f_0, \end{array}\right. } $$

where the collision operator is given in (1.4).

#### Theorem 1

This Cauchy problem is locally well-posed in $\omega ^{\alpha } L^\infty (\mathbb {T})$ if $\alpha > - \frac{5}{4}$. More precisely, if $f_0 \in \omega ^\alpha L^\infty$, there exists a unique solution $f \in \mathcal {C}([0,T],\omega ^\alpha L^\infty )$, where

$$ T \gtrsim \Vert f_0 \Vert _{\omega ^\alpha L^\infty }^{-2}. $$

#### Remark 1

What is the significance of the above theorem? The simplest case is α=0, which simply gives local well-posedness in $L^\infty$. The case α=-1 is important since $\omega ^{-1} L^\infty$ is a function space which includes the singular Rayleigh–Jeans equilibria $\mathfrak {f}_{\beta ,0}$. Finally, the case $\alpha \ne 0$ can be interpreted as the propagation by the nonlinear problem of the vanishing or singular behavior at $p=0 \mod 2\pi$ - related ideas will play an important role in the nonlinear stability questions examined later in this paper. Finally from the last part of the proof, we expect the exponent $-\frac{5}{4}$ to be the sharp one.

#### Proof

We claim that the collision operator is bounded on $\omega ^\alpha L^\infty$. Taking this fact for granted for a moment, the equation can be written via Duhamel’s formula as

$$ f = f_0 + \Phi [f] \quad \text{ where } \quad \Phi [f] = \int _0^t \mathcal {C}[f](s) \, \textrm{d}s, $$

and the mapping Φ can be bounded in $\mathcal {C}([0,T],\omega ^\alpha L^\infty )$ by

$$ \Vert \Phi [f] \Vert _{\mathcal {C}([0,T],\omega ^\alpha L^\infty )} \lesssim T \Vert f \Vert _{\mathcal {C}([0,T],\omega ^\alpha L^\infty )}^3. $$

Local well-posedness is then an easy consequence of the Banach fixed point theorem.

We now turn to proving boundedness of the collision operator in $\omega ^\alpha L^\infty$. The denominator in the integrand in (1.4) is bounded by

$$\begin{aligned} \sqrt{ F_+(p_0,p_2)} = \sqrt{\left[ \cos \left( \frac{p_0}{2}\right) +\cos \left( \frac{p_2}{2}\right) \right] ^2 +4\sin \left( \frac{p_0}{2}\right) \sin \left( \frac{p_2}{2}\right) } \ge 2 \sqrt{\omega _0 \omega _2}, \end{aligned}$$

implying that

3.1 $$\begin{aligned} \begin{aligned} |\mathcal {C}[f](t,p_0)|&\le \int _0^{2\pi } \sqrt{\omega _0 \omega _2} \omega _1 \omega _3 \left| \prod _{\ell =0}^3 f_\ell \left( \frac{1}{f}+\frac{1}{f_1}-\frac{1}{f_2}-\frac{1}{f_3}\right) \right| d p_2. \end{aligned} \end{aligned}$$

We now resort to the inequalities

$$\begin{aligned}&\sqrt{\omega _0 \omega _2} \omega _1 \omega _3 \omega _0^{-\alpha } \prod _{\ell =0}^3 \omega _\ell ^{\alpha } \left( {\omega _0^{-\alpha }}+ {\omega _1^{-\alpha }}+{\omega _2^{-\alpha }}+{\omega _3^{-\alpha }}\right) \lesssim {\left\{ \begin{array}{ll} \omega _2^{\frac{1}{2}+\alpha } &  \text{ if } -1 \le \alpha \le 0 \\ 1 &  \text{ if } \alpha \ge 0. \end{array}\right. } \end{aligned}$$

For the first inequality, when $\alpha \in [-1,0]$, expanding the terms in the bracket we see that $\omega _2^{\frac{1}{2}+\alpha }$ is the worst term and all the rest of the factors are bounded by 1.

The second inequality, when α>0, is a consequence of $\omega _1 \omega _2 \omega _3 \lesssim \omega _0$, see Lemma 19, which in this case implies $(\omega _1 \omega _2 \omega _3)^\alpha \lesssim \omega _0^\alpha$. Indeed by expanding the terms in the bracket, the whole expression becomes $\omega _0^{\frac{1}{2}}\omega _2^{\frac{1}{2}+\alpha } \omega _1^{1+\alpha }\omega _3^{1+\alpha } \big ( {\omega _0^{-\alpha }}+ {\omega _1^{-\alpha }}+{\omega _2^{-\alpha }}+{\omega _3^{-\alpha }}\big )$. The worst term then is the first one, which after using that $(\omega _1 \omega _2 \omega _3)^\alpha \lesssim \omega _0^\alpha$, is bounded by $\omega _0^{\frac{1}{2}} \omega _1 \omega _2^{\frac{1}{2}}\omega _3 \lesssim 1$. All the others are bounded, as there is no negative exponent.

As a consequence,

$$ \Vert \omega ^{-\alpha } \mathcal {C}[f] \Vert _{ L^\infty } \lesssim \Vert \omega ^{-\alpha } f \Vert _{L^\infty }^3 \int _0^{2\pi } \max (1,\omega _2^{\frac{1}{2} + \alpha }) \, \textrm{d}p_2 \lesssim \Vert \omega ^{-\alpha } f \Vert _{L^\infty }^3, $$

since this integral is finite whenever $\alpha > -\frac{3}{2}$, which is always the case since $\alpha \ge -1$.

In fact, using the explicit parametrisation of the resonant manifold, (1.2), which we recall is

$$\begin{aligned} p_1= &   h(p_0,p_2),\quad p_3 = p_0+h-p_2, \quad h(p_0,p_2) = \frac{p_2-p_0}{2}\\  &   \quad + 2 \arcsin \left( \tan \frac{|p_2-p_0|}{4} \cos \frac{p_2+p_0}{4} \right) , \end{aligned}$$

we can sharpen the threshold for α. Namely, we will show that

$$ Q_\alpha (p_0,p_2)\!:=\! \sqrt{\omega _0 \omega _2} \omega _1 \omega _3 \prod _{\ell =1}^3 \omega _\ell ^{\alpha } \left( {\omega _0^{-\alpha }}\!+\! {\omega _1^{-\alpha }}\!+\!{\omega _2^{-\alpha }}\!+\!{\omega _3^{-\alpha }}\right) \!\lesssim \! \max (1,\omega _2^{\frac{3}{2} + 2\alpha }).$$

We focus on the region where both $p_0, p_2$ are close to 0. In particular, due to symmetry, we treat only the case $0<p_2<p_0 \ll 1$. We Taylor expand *h*, around (0, 0) from which we obtain the asymptotics

$$\begin{aligned} h(p_0,p_2)&= -\frac{1}{16} p_0p_2 (p_0-p_2) + \mathcal {O}\Big ((p_0-p_2)^3(p_0+p_2)^2\Big ), \\ p_3&= (p_0-p_2) - \frac{1}{16} p_0p_2 (p_0-p_2) + \mathcal {O}\Big ((p_0-p_2)^3(p_0+p_2)^2\Big ). \end{aligned}$$

In this region then, up to leading order, $\omega _0 \sim p_0, \omega _2\sim p_2$, $\omega _1\sim p_0p_2 (p_0-p_2)$ and $\omega _3 \sim p_0-p_2$. We turn then to our quantity $Q_\alpha$ which is the sum of four terms and we estimate each term separately (for α<0):

$$\begin{aligned}&\omega _0^{\frac{1}{2}-\alpha } \omega _2^{\frac{1}{2}+\alpha } \omega _1^{1+\alpha } \omega _3^{1+\alpha } \sim p_0^{\frac{3}{2}}p_2^{\frac{3}{2} + 2\alpha } (p_0-p_2)^{2+2\alpha }\\&\omega _0^{\frac{1}{2}} \omega _2^{\frac{1}{2}+\alpha } \omega _3^{1+\alpha }\omega _1 \sim p_0^{\frac{3}{2}}p_2^{\frac{3}{2} + \alpha } (p_0-p_2)^{2+\alpha }\\&\omega _0^{\frac{1}{2}} \omega _2^{\frac{1}{2}} \omega _3^{1+\alpha }\omega _1^{1+\alpha }\sim p_0^{\frac{3}{2}+ \alpha }p_2^{\frac{3}{2} + \alpha } (p_0-p_2)^{2+2\alpha } \\&\omega _0^{\frac{1}{2}} \omega _2^{\frac{1}{2}+ \alpha } \omega _3 \omega _1^{1+\alpha }\sim p_0^{\frac{3}{2}+ \alpha }p_2^{\frac{3}{2} + 2 \alpha } (p_0-p_2)^{2+\alpha }. \end{aligned}$$

I indeed, if α<0, the worst exponent in $Q_\alpha$ is $p_2^{\frac{3}{2} + 2 \alpha }$. While when $\alpha \ge 0$, the quantity is upper bounded by 1, as written above.

When $p_0,p_2$ are far from the edges, or when one of them is, the same (or simpler) bounds hold.

Finally,

$$ \Vert \omega ^{-\alpha } \mathcal {C}[f] \Vert _{ L^\infty } \lesssim \Vert \omega ^{-\alpha } f \Vert _{L^\infty }^3 \int _0^{2\pi } \max (1,\omega _2^{\frac{3}{2} + 2 \alpha }) \, \textrm{d}p_2 \lesssim \Vert \omega ^{-\alpha } f \Vert _{L^\infty }^3, $$

since this integral is finite if and only if $\alpha > -\frac{5}{4}$.

Note that these calculations indicate that the threshold $\alpha > -\frac{5}{4}$, is in fact the optimal one for the local well-posedness in $\omega ^{\alpha } L^\infty (\mathbb {T})$. □

### Unboundedness in weighted $L^p$ spaces for p<3

In the preceding subsection, we saw that local well-posedness in weighted $L^\infty$ spaces was a consequence of the boundedness of the collision operator in these spaces. In the present section, we show that the collision operator cannot be bounded on $L^p$-type spaces for p<3. This does not imply ill-posedness, but this shows that well-posedness can only be the result of a very delicate nonlinear mechanism.

From now on we will write

$$ \underline{h}(x,z) = x-z + h(x,z) = \frac{x-z}{2} + 2 \arcsin \left( \tan \frac{|z-x|}{4} \cos \frac{z+x}{4} \right) \mod 2\pi . $$

#### Theorem 2

The collision operator $\mathcal {C}$ is not bounded on $L^p(\mathbb {T})$ if p<3.

#### Proof

Three specific points. Given $x_0$, we choose $z_0$ such that $z \mapsto h(x_0,z)$ reaches its global maximum at $z_0$ (this maximum is unique modulo 2π for almost all values of $x_0$). It follows from this choice that $\underline{h}(x_0,z_0) = z_0$ (modulo 2π). Indeed, there holds by symmetry between $p_2$ and $p_3$: $h(x_0,z_0) = h(x_0, \underline{h}(x_0,z_0))$; since $z \mapsto h(x_0,z)$ reaches its global maximum at $z_0$, this implies that $\underline{h}(x_0,z_0) = z_0$.

We set then

$$ p_0^0 = x_0, \quad p_1^0 = h(x_0,z_0), \quad p_2^0 = z_0, \quad p_3^0 = \underline{h}(x_0,z_0) = p_2^0. $$

We claim that we can choose $x_0$ such that

1. The numbers $p^0_0,p^0_1, p^0_2$ are distinct and different from 0 (modulo 2π).
2. If the $(p_i)$ belong to the resonant manifold, meaning that $p_3 = p_0 + p_1 - p_2$ and $p_1 = h(p_0,p_2)$, where *h* is given by (1.2), if furthermore $p_0 = p_0^0$ and if finally $(p_2,p_3) = (p^0_{i_2},p^0_{i_3})$ for some $\{i_2,i_3\} \in \{0,1,2 \}$, then $(p_1,p_2,p_3) = (p_1^0,p_2^0,p_3^0)$.

It is easiest to give a concrete example and to choose $x_0 =2$ and $z_0$ to be the local maximum of $z \mapsto h(x_0,z)$, or in other words,

$$ p_0^0 = 2, \quad p_1^0 = 1.184..., \quad p_2^0 = 4.733.... $$

We now fix $p_0 = p_0^0 = 2$, and try all combinations of $i_2,i_3$ as in property (2) above

- If $$p_2 = p^0_0$$, then $$p_3 = \underline{h}(p_0^0,p_0^0) = 0$$.
- If $$p_2 = p^0_1$$, then $$p_3 = \underline{h}(p_0^0,p_0^1) = 0.698...$$.
- If $p_2 = p_2^0$, then $p_1= h(p_0^0,p_2^0) = p_1^0$ and $p_3 = \underline{h}(p_0^0,p_2^0) = p_2$ modulo 2π.

The example giving unboundedness of the collision operator. We will test the collision operator on the following family of functions, which are uniformly bounded in $L^p$:

$$ f^\epsilon = \epsilon ^{-\frac{2}{p}} \textbf{1}_{[p_0^0,p_0^0+\epsilon ^2]} + \epsilon ^{-\frac{2}{p}} \textbf{1}_{[p^0_1,p^0_1+\epsilon ^2]} + \epsilon ^{-\frac{1}{p}} \textbf{1}_{[p^0_2,p^0_2+\epsilon ]}. $$

We will denote *K* as the integration kernel in the collision operator; it only vanishes if $p_i = 0$ for some i=1,2,3,4. The collision operator can be naturally split into a positive and a negative part

$$\begin{aligned} \mathcal {C}[f](p_0)&= \int _0^{2\pi } K(p_0,p_2)\prod _{\ell =0}^3 f_\ell ^\epsilon \left( \frac{1}{f^\epsilon }+\frac{1}{f_1^\epsilon }-\frac{1}{f_2^\epsilon }-\frac{1}{f_3^\epsilon }\right) \,dp_2 \\&= \mathcal {C}_+[f](p_0) - \mathcal {C}_-[f](p_0), \end{aligned}$$

where it is understood that the variables $(p_0,p_1,p_2,p_3)$ in the integral are parameterized as $(p_0,h(p_0,p_2),p_2,\underline{h}(p_0,p_2))$.

On the one hand, if $|p_0-p_0^0| \ll \epsilon ^2$, it follows from property (2) above that

$$\begin{aligned} \mathcal {C}_+[f^\epsilon ](p_0)&= \int _0^{2\pi } K(p_0,p_2) \left[ f^\epsilon _1 f^\epsilon _2 f^\epsilon _3 + f^\epsilon _0 f^\epsilon _2 f^\epsilon _3 \right] \,dp_2 \\&\lesssim \epsilon ^{-\frac{4}{p}} \int _0^{2\pi } \left[ \textbf{1}_{[p_1^0,p_1^0+\epsilon ^2]} (p_1) \textbf{1}_{[p_2^0,p_2^0+\epsilon ]}(p_2) \textbf{1}_{[p_2^0,p_2^0+\epsilon ]}(p_3) \right. \\&\qquad \qquad \qquad \qquad \left. + \textbf{1}_{[p_0^0,p_0^0+\epsilon ^2]}(p_0) \textbf{1}_{[p_2^0,p_2^0+\epsilon ]}(p_2) \textbf{1}_{[p_2^0,p_2^0+\epsilon ]}(p_3) \right] \,dp_2 \\&\lesssim \epsilon ^{1 - \frac{4}{p}}. \end{aligned}$$

On the other hand, still assuming that $|p_0-p_0^0| \ll \epsilon ^2$,

$$\begin{aligned} \mathcal {C}_-[f^\epsilon ](p_0)&\ge \int _0^{2\pi } K(p_0,p_2) f^\epsilon _0 f^\epsilon _1 f^\epsilon _2 \,dp_2 \\&= \epsilon ^{-\frac{5}{p}} \textbf{1}_{[p_0^0,p_0^0+\epsilon ^2]} (p_0) \int _0^{2\pi } K(p_0,p_2) \textbf{1}_{[p^0_1,p^0_1+\epsilon ^2]} (p_1) \textbf{1}_{[p^0_2,p^0_2+\epsilon ]} (p_2) \,dp_2 \\&\ge \epsilon ^{1 - \frac{5}{p}}. \end{aligned}$$

Overall, we get that that, if $|p_0 - p_0^0| \ll \epsilon ^2$,

$$ \left| \mathcal {C}[f^\epsilon ](p_0) \right| \gtrsim \epsilon ^{1 - \frac{5}{p}}. $$

This implies that

$$ \Vert \mathcal {C}[f^\epsilon ] \Vert _{L^p} \gtrsim \epsilon ^{1 - \frac{3}{p}}, $$

from which the desired result follows since $\Vert \mathcal {C}[f^\epsilon ] \Vert _{L^p} \rightarrow \infty$ as $\epsilon \rightarrow 0$ if p<3.

□

## First properties of the linearized operator

Recall that the Rayleigh–Jeans equilibria are given by

$$ \mathfrak {f}(p) = \mathfrak {f}_{\beta , \gamma } (p) = \frac{1}{\beta \omega (p) + \gamma }, \qquad \beta ,\gamma >0.$$

We now linearise around $\mathfrak {f}(p)$: If $f(t,p) = \mathfrak {f}(p) (1+ g(t,p))$, then *g*(*t*, *p*) satisfies the following equation

4.1 $$\begin{aligned} \begin{aligned}&\partial _tg(t,p) = \mathfrak {f}(p)^{-1} \int _{\mathbb {T}^3} \delta (\Sigma ) \delta (\Omega ) \left[ \prod _{\ell =0}^3 \omega _\ell \mathfrak {f}(p_\ell ) \right] \times \\&\prod _{\ell =0}^3 (1+ g(t,p_\ell ) ) \left( \frac{1- g(t,p)}{\mathfrak {f}(p)}+\frac{1- g(t,p_1)}{\mathfrak {f}(p_1)}-\frac{1- g(t,p_2)}{\mathfrak {f}(p_2)}-\frac{1- g(t,p_3)}{\mathfrak {f}(p_3)}\right) \,\, \textrm{d}p_{1,2,3} + \mathcal {O}(g^2)\\&\quad = [L g](k) + \mathcal {O}(g^2), \end{aligned} \end{aligned}$$

where

$$ [L g](p) = \frac{1}{\mathfrak {f}(p)} \int _{\mathbb {T}^3} \delta (\Sigma ) \delta (\Omega ) \left[ \prod _{\ell =0}^3 \omega _\ell \mathfrak {f}_\ell \right] \left[ -\frac{g}{\mathfrak {f}}-\frac{g_1}{\mathfrak {f}_1}+\frac{g_2}{\mathfrak {f}_2}+\frac{g_3}{\mathfrak {f}_3}\right] \,\, \textrm{d}p_{1,2,3}. $$

The operator *L* is symmetric in $L^2$ and the Dirichlet form is positive, namely,

$$ \langle -L g,g \rangle = \frac{1}{4} \int \delta ({\Sigma })\delta ({\Omega }) \left[ \prod _{\ell =0}^3 \omega _\ell \mathfrak {f}_\ell \right] \left[ \frac{g_3}{\mathfrak {f}_3}+ \frac{g_2}{\mathfrak {f}_2} - \frac{g}{\mathfrak {f}} - \frac{g_1}{\mathfrak {f}_1}\right] ^2 \, \textrm{d}p_{1,2,3} \ge 0.$$

We split the linearised operator *L* into L=-A+K, where *A* is the multiplication operator

$$ [Ag](p)= a(p) g(p), \qquad a(p) = \frac{\omega (p)}{\mathfrak {f}(p)}\int _{I^3} \delta (\Sigma ) \delta (\Omega ) \left[ \prod _{\ell =1}^3 \omega _\ell \mathfrak {f}_\ell \right] \, \textrm{d}p_{1,2,3}, $$

and *K* is the integral operator

$$ K = - K_1 + 2 K_2, \qquad {\left\{ \begin{array}{ll} &  \displaystyle [K_1 g](p) = \frac{1}{\mathfrak {f}(p)} \int \delta (\Sigma ) \delta (\Omega ) \left[ \prod _{\ell =0}^3 \omega _\ell \mathfrak {f}_\ell \right] \frac{g_1}{\mathfrak {f}_1} \, \, \textrm{d}p_{1,2,3}\\ &  \displaystyle [K_2 g](p) = \frac{1}{\mathfrak {f}(p)} \int \delta (\Sigma ) \delta (\Omega ) \left[ \prod _{\ell =0}^3 \omega _\ell \mathfrak {f}_\ell \right] \frac{g_2}{\mathfrak {f}_2} \, \, \textrm{d}p_{1,2,3}. \end{array}\right. } $$

One checks that $K_1$ and $K_2$ are symmetric operators.

With the help of the technical Lemmas 14 and 13, the multiplier *a* and the kernels $K_1$ and $K_2$ can be written as (we always abuse notations and identify an operator and its kernel)

4.2 $$\begin{aligned} \begin{aligned}&a(p) = \frac{\omega (p)}{\mathfrak {f}(p)} \int _0^{2\pi } \omega _1 \omega _2 \omega _3 \mathfrak {f}_1 \mathfrak {f}_2 \mathfrak {f}_3 \frac{\, \textrm{d}p_2}{\sqrt{ F_+(p,p_2)}}, \qquad p_1 = h(p,p_2),\; p_3 = p + p_1 - p_2 \\&K_1(p,p_1) = \frac{\textbf{1}_{F_-(p,p_1)>0}}{\sqrt{F_-(p,p_1)}}\omega \omega _1 \omega _2 \omega _3 \mathfrak {f}_2 \mathfrak {f}_3, \qquad p_1 = h(p,p_2),\; p_3 = p + p_1 - p_2 \\&K_2(p,p_2) = \frac{1}{\sqrt{F_+(p,p_2)}} \omega \omega _1 \omega _2 \omega _3 \mathfrak {f}_1 \mathfrak {f}_3, \qquad p_1 = h(p,p_2),\; p_3 = p + p_1 - p_2, \end{aligned} \end{aligned}$$

where $F_{\pm }$ are defined in (1.5).

The next four lemmas lay out the basic properties of the linear operators $L,A,K_1,K_2$. These results already appear in [11], except for the case $\gamma \ne 0$ of Lemma 2.

### Lemma 1

(Kernel of the linearized operator) The kernel of *L* is spanned by $\mathfrak {f}$ and $\omega \mathfrak {f}$.

### Proof

This follows immediately from the characterization of collisional invariants, Theorem 2.2 in [11]. □

### Lemma 2

(Asymptotics of the multiplier function) For any $\beta ,\gamma >0$,

$$ a(p) \sim _{\beta ,\gamma } \sin \left( \frac{p}{2} \right) ^{\frac{5}{3}}. $$

### Proof

This result is proven in Lemmas 16 and 18, for zero and non-zero mass, respectively. □

### Lemma 3

(Compactness of the weighted $K_2$ operator) For any β, γ, the operator $a^{-\frac{1}{2}} K_2 a^{-\frac{1}{2}}$ is compact on $L^2$.

### Proof

Using Lemma 2 and the fact that $\sqrt{F_+} \ge \sqrt{\omega _0 \omega _2}$, we find that

$$ a(p)^{-\frac{1}{2}} K_2(p,p_2) a(p_2)^{-\frac{1}{2}} \lesssim \sin \left( \frac{p}{2} \right) ^{-\frac{1}{3}} \sin \left( \frac{p_2}{2} \right) ^{- \frac{1}{3}}, $$

so that the corresponding operator is Hilbert-Schmidt, and therefore compact on $L^2$. □

### Lemma 4

(Compactness of the weighted $K_1$ operator) For any β, γ, the operator $a^{-\frac{1}{2}} K_1 a^{- \frac{1}{2}}$ is compact on $L^2$.

### Proof

Let $\widetilde{K}_1 = a^{-\frac{1}{2}} K_1 a^{- \frac{1}{2}}$. We will decompose this operator into

$$\begin{aligned}&\widetilde{K}_1(p,p_1) = \widetilde{K}_1^{(1)}(p,p_1) + \widetilde{K}_1^{(2)}(p,p_1) + \widetilde{K}_1^{(3)}(p,p_1),\\&\widetilde{K}_1^{(1)}(p,p_1) = \mathbb {1}_{\min (\omega ,\omega _1)< \delta } \widetilde{K}_1(p,p_1) \\&\widetilde{K}_1^{(2)}(p,p_1) = \mathbb {1}_{\begin{array}{c} \min (\omega ,\omega _1) > \delta \\ F_-(p,p_1) < \epsilon \end{array}} \widetilde{K}_1(p,p_1), \end{aligned}$$

where $\epsilon , \delta >0$ may vary. We claim that

4.3 $$\begin{aligned}&\Vert \widetilde{K}_1^{(1)} \Vert _{L^2 \rightarrow L^2} \lesssim \delta ^{\frac{1}{6}}, \qquad \Vert \widetilde{K}_1^{(2)} \Vert _{L^2 \rightarrow L^2} \lesssim \left( \frac{\epsilon }{\delta }\right) ^{\frac{1}{6}} \quad \text{ and } \end{aligned}$$

4.4 $$\begin{aligned}&\quad \Vert \widetilde{K}_1^{(3)}(p,p_1) \Vert _{L^\infty _{p,p_1}} := \text {ess sup}_{(p,p_1)\in \mathbb {T}^2} | \widetilde{K}_1^{(3)}(p,p_1) |\lesssim _{\epsilon ,\delta } 1. \end{aligned}$$

These bounds imply that $\widetilde{K}_1^{(3)}$ is compact for any $\epsilon ,\delta$, and that $\widetilde{K}_1$ can be approximated by compact operators in the operator norm. By closedness of the class of compact operators, this implies that $\widetilde{K}_1$ is compact.

There remains to prove the bounds on the operator norms of $\widetilde{K}_1^{(1)}$ and $\widetilde{K}_1^{(2)}$. We will rely on the following variant of Schur’s test: given a weight *w* and a symmetric operator *S* with kernel *S*(*x*, *y*),

$$ \Vert S \Vert _{L^2 \rightarrow L^2} \lesssim \sup _x w(x) \int |S(x,y)| \frac{\, \textrm{d}y}{w(y)}. $$

Before applying this lemma, we record the following estimate for $\widetilde{K}_1$, which follows from Lemma 15: if $p_1'(p) < p_1''(p)$ are the two zeros of the function $p_1\mapsto F_-(p,p_1)$, for fixed $p\in (0,2\pi )$ (that we get from Lemma 15),

$$ \text{ if } p_1 \in (0, p_1'(p)), \qquad |\widetilde{K}_1|(p,p_1) \lesssim (p_1'-p_1)^{-\frac{1}{2}} \omega ^{-\frac{1}{3}} \omega _1^{\frac{1}{6}}. $$

There is a symmetric estimate for $p_1 \in (p_1'',2\pi )$, but in order to make notations lighter, we will restrict the value of $p_1$ to $p_1 \in (0, p_1'(p))$. Then the variant of Schur’s test above with the weight $w = \omega ^{\frac{1}{2}}$ gives

$$\begin{aligned} \Vert \widetilde{K}_1^{(1)} \Vert _{L^2 \rightarrow L^2}&\lesssim \sup _p \int _0^{p_1'} (p_1'-p_1)^{-\frac{1}{2}} \omega ^{\frac{1}{6}} \omega _1^{-\frac{1}{3}} \mathbb {1}_{\min (\omega ,\omega _1)< \delta } \,\, \textrm{d}p_1 \\&\lesssim \sup _{\omega < \delta } \int _0^{p_1'} (p_1'-p_1)^{-\frac{1}{2}} \omega ^{\frac{1}{6}} \omega _1^{-\frac{1}{3}} \,\, \textrm{d}p_1 \\&+ \sup _p \int _0^{\min (\delta ,p_1')} (p_1'-p_1)^{-\frac{1}{2}} \omega ^{\frac{1}{6}} \omega _1^{-\frac{1}{3}} \,\, \textrm{d}p_1 \lesssim \delta ^{\frac{1}{6}}, \end{aligned}$$

where the last inequality is a consequence of Hölder’s inequality.

Turning to $\widetilde{K}_1^{(2)}$, there holds on the support of its kernel $|p_1 - p_1'| \lesssim \frac{\epsilon }{\omega } \lesssim \frac{\epsilon }{\delta }$. Therefore,

$$\begin{aligned} \Vert \widetilde{K}_1^{(2)} \Vert _{L^2 \rightarrow L^2}&\lesssim \sup _p \int _{\max (0,p_1'-C \frac{\epsilon }{\delta })}^{p_1'} (p_1'-p_1)^{-\frac{1}{2}} \omega ^{\frac{1}{6}} \omega _1^{-\frac{1}{3}} \,\, \textrm{d}p_1 \lesssim \left( \frac{\epsilon }{\delta }\right) ^{\frac{1}{6}}, \end{aligned}$$

which concludes the proof. □

Combining these four lemmas, we will obtain a crucial lower bound on the linearized operator.

### Proposition 4.1

For any $\beta ,\gamma >0$, we have for all $g \in L^2$ such that $g \perp _{L^2_a} \operatorname {Ker} L$,

$$\begin{aligned} \langle -L g,g\rangle \gtrsim \int a(p) |g(p)|^2 \, \textrm{d}p. \end{aligned}$$

### Proof

Let $$\widetilde{K} = a^{-\frac{1}{2}} K a^{- \frac{1}{2}}$$. Then

$$\begin{aligned} 0 \le - \langle L g,g \rangle = \langle A g,g \rangle - \langle K g,g \rangle&= \langle a^{\frac{1}{2}} g, a^{\frac{1}{2}} g \rangle - \langle \widetilde{K} (a^{\frac{1}{2}} g), (a^{\frac{1}{2}} g) \rangle \\&= \langle (\operatorname {Id} - \widetilde{K}) a^{\frac{1}{2}} g ,a^{\frac{1}{2}} g \rangle . \end{aligned}$$

Since $\widetilde{K}$ is compact and self-adjoint, its spectrum is discrete away from 0. It follows from the above formula that $\widetilde{K}$ cannot have eigenvalues >1, due to the positive sign of the Dirichlet form, and that the eigenspace associated to the eigenvalue 1 of $\widetilde{K}$ coincides with the space $a^{\frac{1}{2}}\operatorname {Ker} L$, i.e. that $\operatorname {Ker} (I - \widetilde{K}) = a^{\frac{1}{2}}\operatorname {Ker} L$. Indeed,

$$\begin{aligned} \widetilde{K}f = f \iff a^{-\frac{1}{2}} K a^{-\frac{1}{2}} f=f \iff Kg=Ag \iff L g=0, \end{aligned}$$

with $f =a^{\frac{1}{2}} g$, where we stress that *L* is acting on the original variable *g*, while $\widetilde{K}$ acts on the conjugated variable $a^{\frac{1}{2}}g$, in flat $L^2$.

Finally, if $f =a^{\frac{1}{2}} g \in \operatorname {Ker}(\operatorname {Id} - \widetilde{K})^\perp$, then due to spectral theorem $\langle (\operatorname {Id} - \widetilde{K})f,f \rangle \ge \delta \Vert f \Vert _{L^2}^2$ for some δ>0, and thus

$$ - \langle L g,g \rangle = \langle A g,g \rangle - \langle \widetilde{K} a^{\frac{1}{2}} g, a^{\frac{1}{2}} g \rangle \gtrsim \int a(p) |g(p)|^2 \, \textrm{d}p. $$

□

Turning to the evolution problem, we note first that it admits a unique solution for $L^2$ data.

### Lemma 5

(Existence and uniqueness) For $g_0 \in L^2$, there exists a unique solution $g(t,p) \in \mathcal {C}([0,\infty ],L^2)$ to the Cauchy problem

$$ {\left\{ \begin{array}{ll} \partial _t g - Lg = 0\\ g(t=0) = g_0 \end{array}\right. } $$

which we will denote $g(t) = e^{tL} g_0$. Furthermore, its $L^2$ norm is bounded by that of the data:

$$ \text{ for } \text{ any } t \ge 0, \qquad \Vert g(t) \Vert _{L^2} \le \Vert g_0 \Vert _{L^2}. $$

### Proof

We saw that $a^{-\frac{1}{2}} K_1 a^{-\frac{1}{2}}$ and $a^{-\frac{1}{2}} K_2 a^{-\frac{1}{2}}$ are bounded on $L^2$, hence this is also the case for $K_1$ and $K_2$. Therefore, *L* is bounded and the lemma follows by a fixed point theorem. That the $L^2$ norm is non-increasing follows from a simple $L^2$ energy estimate, since *L* is dissipative, $\langle Lg,g\rangle \le 0$. □

As a consequence of the lower bound proved in the previous proposition, we get the following corollary:

### Corollary 1

(Dissipation inequality) For $g_0 \in L^2 \cap \big ( \operatorname {Ker} L\big )^{\perp _a}$,

$$ \int _0^\infty \int a(p) |e^{tL} g_0(p)|^2 \, \textrm{d}p \, \textrm{d}t \lesssim \Vert g_0 \Vert _{L^2}^2. $$

This corollary quantifies time decay for the solution, but it will be insufficient for our purposes. Indeed, the equation is ill-posed in weighted $L^2$ spaces, so that this topology cannot be used to control nonlinear terms.

In the next section, we aim to obtain pointwise decay, which will correct this shortcoming.

## Pointwise decay for the linearized operator

In all that follows we assume that the chemical potential γ>0, that is we study the linearised operator around non-singular RJ equilibria.

In this section we investigate how the energy dissipation leads to a polynomially fast relaxation for the linearized semigroup, pointwise and away from the edges. To this purpose we explore how the edges of the domain, where the weight in the Poincaré Inequality of the previous section vanishes, interact with the bulk of the domain, where the weight is lower-bounded.

### Energy decay in the bulk

We define that

$$ \langle t \rangle = 10 + |t|, $$

as well as the following subintervals of [0,2π], corresponding to the edges and the bulk of the domain, respectively:

5.1 $$\begin{aligned} \begin{aligned}&\mathcal {E}_{t,\alpha } = \{ p \in [0,2\pi ]: p< \langle t \rangle ^{-\alpha },\ p> 2\pi -\langle t \rangle ^{-\alpha } \} \text { and}\\&\mathcal {B}_{t,\alpha } = \{ p \in [0,2\pi ]: \langle t \rangle ^{-\alpha } \le p \le 2\pi - \langle t \rangle ^{-\alpha }\}. \end{aligned} \end{aligned}$$

We now define the following functionals:

5.2 $$\begin{aligned} \begin{aligned} m(t) = \int _{\mathcal {B}_{t,\alpha }} |g(t,p)|^2 d p, \quad n(t) = \int _{\mathcal {E}_{t,\alpha } }|g(t,p)|^2 d p, \quad q(t) = \sup _{p\ \in \ \mathcal {E}_{t,\alpha } } |g(t,p)|. \end{aligned} \end{aligned}$$

#### Lemma 6

Assume $g_0 \in L^2 \cap \big ( \operatorname {Ker}L\big )^\perp$, let $\alpha < \frac{3}{5}$, and suppose that

$$ m(0)+n(0) \le 1 \qquad \text{ and } \qquad q(t) \le \langle t \rangle ^{e}\quad \text { for all } t \ge 0, $$

where $$e \in \mathbb {R}$$. Then

$$ m(t) \lesssim _\alpha \langle t \rangle ^{2e - \alpha }. $$

#### Proof

We consider solutions with initial data $g_0 \in (\operatorname {Ker} L)^\perp$, which is propagated by the linear flow, so that $g_t \in (\operatorname {Ker} L)^\perp$ for t>0.

We decompose $g_t$ into its projection on $\operatorname {Ker} L$ with respect to the weighted inner product $\langle \cdot , \cdot \rangle _a$ and the orthogonal component as

$$\begin{aligned} g_t = g^{\parallel }_t + g_t^{\perp _a}, \end{aligned}$$

where

$$ g_t^{\parallel } \in \operatorname {Ker} L \quad \text{ and } \quad \int g_t^{\perp _a} a \phi _i \, \textrm{d}p=0, \ \quad \text { for } \ \phi _i \in \operatorname {Ker} L, \quad \ i=1,2.$$

The elements of the two-dimensional $\operatorname {Ker} L$ are given in Lemma 1: $\phi _1=\mathfrak {f}$, $\phi _2=\mathfrak {f}\omega$. By symmetry of *L* and the fact that $L g_t^{\parallel } =0$, we have, for all *t*,

$$\begin{aligned} -\langle Lg_t, g_t \rangle = - \langle L g_t^{\perp _a}, g_t^{\perp _a} \rangle . \end{aligned}$$

This implies, together with Lemma 2 and Proposition 4.1 for $g_t^{\perp _\alpha }$,

5.3 $$\begin{aligned} \begin{aligned} -\frac{d}{dt} (m(t) + n(t)) = - 2\int g(t,p) L g(t,p) \, \textrm{d}p&= - 2\int g^{\perp _a}(t,p) L g^{\perp _a}(t,p) \, \textrm{d}p\\&\gtrsim \int a(p) |g^{\perp _a}(t,p)|^2 \, \textrm{d}p \\&\gtrsim \int _{\mathcal {B}_{t,\alpha }} \omega (p)^{\frac{5}{3}} |g^{\perp _a}(t,p)|^2 \, \textrm{d}p. \end{aligned} \end{aligned}$$

We will now lower-bound the right-hand side by a quantity depending on *m* and *n*.

We start with the decomposition

$$ g_t^{\parallel } = c_1(t)\phi _1 + c_2(t)\phi _2, \qquad c_1(t), c_2(t) \in \mathbb {R} $$

and the fact that, since $g_t \in (\operatorname {Ker} L)^\perp$,

$$0=\langle g_t, \phi _i \rangle = \int _{\mathcal {B}_{t,\alpha }}(g_t^{\perp _a} + g_t^\parallel ) \phi _i \, \textrm{d}p + \int _{\mathcal {E}_{t,\alpha }} g_t \phi _i \, \textrm{d}p, \ \quad i=1,2, $$

which, after rearranging, gives

5.4 $$\begin{aligned} - \int _{\mathcal {B}_{t,\alpha }} g_t^{\perp _a} \phi _i - \int _{\mathcal {E}_{t,\alpha }} g_t \phi _i = \int _{\mathcal {B}_{t,\alpha }} [c_1(t) \phi _1 + c_2(t) \phi _2]\ \phi _i, \ \quad i =1,2. \end{aligned}$$

This can be written in a matrix equation form where the right-hand side is $G_{\mathcal {B}_{t,\alpha }}(c_1(t), c_2(t))^T$, with $G_{\mathcal {B}_{t,\alpha }}$ being the 2×2 matrix with entries

$$\begin{aligned} (G_{\mathcal {B}_{t,\alpha }})_{i,j=1}^2 = \int _{\mathcal {B}_{t,\alpha }}\phi _i \phi _j. \end{aligned}$$

Now observe that since $\phi _1, \phi _2$ are linearly independent, the matrix with entries $(G_{\mathbb {T}})_{i,j=1}^2 = \int _{\mathbb {T}}\phi _i \phi _j$ is invertible. Since $\mathcal {B}_{t,\alpha } \uparrow \mathbb {T}$ as $t \rightarrow \infty$, for times *t* large enough, the Gram matrix $G_{\mathcal {B}_{t,\alpha }}$ approximates the Gram matrix $G_{\mathbb {T}}$ and thus it is also invertible. We leave the details of this claim for the end of the proof.

Thus, we may solve (5.4) for the coefficients $c_1(t), c_2(t)$ and bound them as

$$c(t): = [c_1(t), c_2(t)]^T = - G_{\mathcal {B}_{t,\alpha }}^{-1} \begin{bmatrix} \int _{\mathcal {B}_{t,\alpha }} g_t^{\perp _a} \phi _1 + \int _{\mathcal {E}_{t,\alpha }} g_t \phi _1\\ \int _{\mathcal {B}_{t,\alpha }} g_t^{\perp _a} \phi _2 + \int _{\mathcal {E}_{t,\alpha }} g_t \phi _2, \end{bmatrix}, $$

from which we get, with the help of the Cauchy-Schwarz inequality,

5.5 $$\begin{aligned} |c(t)| \lesssim \Vert G_{\mathcal {B}_{t,\alpha }}^{-1} \Vert \left( \left| \begin{bmatrix} \int _{\mathcal {B}_{t,\alpha }} g_t^{\perp _a} \phi _1\\ \int _{\mathcal {B}_{t,\alpha }} g_t^{\perp _a} \phi _2 \end{bmatrix}\right| + \left| \begin{bmatrix} \int _{\mathcal {E}_{t,\alpha }} g_t \phi _1 \\ \int _{\mathcal {E}_{t,\alpha }} g_t \phi _2 \end{bmatrix}\right| \right) \lesssim \Vert g_t^{\perp _a}\Vert _{L^2(\mathcal {B}_{t,\alpha })} + \sqrt{n(t)\langle t \rangle ^{-\alpha }}. \end{aligned}$$

Since $\Vert G_{\mathcal {B}_{t,\alpha }}^{-1} \Vert , \phi _1, \phi _2$ are bounded (recall γ>0) by the boundedness of $\phi _1, \phi _2$, this allows us to estimate $\Vert g_t^\parallel \Vert _{L^2(\mathcal {B}_{t,\alpha })}$:

$$ \Vert g_t^\parallel \Vert _{L^2(\mathcal {B}_{t,\alpha })}\lesssim |c(t)| \lesssim \Vert g_t^{\perp _a}\Vert _{L^2(\mathcal {B}_{t,\alpha })} + \sqrt{n(t)\langle t \rangle ^{-\alpha }}. $$

This, eventually, we control the whole $\Vert g_t\Vert _{L^2(\mathcal {B}_{t,\alpha })} = \sqrt{m(t)}$ norm:

$$ \sqrt{m(t)} = \Vert g_t\Vert _{L^2(\mathcal {B}_{t,\alpha })} \lesssim \Vert g_t^{\perp _a}\Vert _{L^2(\mathcal {B}_{t,\alpha })} + \Vert g_t^{\parallel }\Vert _{L^2(\mathcal {B}_{t,\alpha })} \lesssim \Vert g_t^{\perp _a}\Vert _{L^2(\mathcal {B}_{t,\alpha })} + \sqrt{n(t)\langle t \rangle ^{-\alpha }}. $$

Squaring both sides,

$$ m(t) \lesssim \Vert g_t^{\perp _a}\Vert _{L^2(\mathcal {B}_{t,\alpha })}^2 + n(t)\langle t \rangle ^{-\alpha } \quad \Rightarrow \quad \Vert g_t^{\perp _a}\Vert _{L^2(\mathcal {B}_{t,\alpha })}^2 \ge C_0 m(t) - n(t)\langle t \rangle ^{-\alpha } $$

for a constant $$C_0$$.

We are now ready to go back to our ODE (5.3). We use on the one hand the above inequality and on the other the fact that

$$ n(t) \lesssim q(t)^2 \langle t \rangle ^{-\alpha } \lesssim \langle t \rangle ^{2e-\alpha }; $$

this gives

$$\begin{aligned} \begin{aligned} -\frac{d}{dt} (m(t) + n(t)) \!\gtrsim \! \int _{\mathcal {B}_{t,\alpha }} \omega (p)^{\frac{5}{3}} |g^{\perp _a}(t,p)|^2 \, \textrm{d}p&\gtrsim C_0 \langle t \rangle ^{- \frac{5\alpha }{3}} \Big ( m(t) \!-\! n(t) \langle t \rangle ^{-\alpha } \Big )\\&\gtrsim C_0 \langle t \rangle ^{- \frac{5\alpha }{3}} m(t) - \langle t \rangle ^{- \frac{8 \alpha }{3}+ 2e}, \end{aligned} \end{aligned}$$

since $\langle t \rangle ^{- 11 \alpha /3} \lesssim \langle t \rangle ^{- 8 \alpha /3}$. Rearranging the ODE we need to solve

5.6 $$\begin{aligned} \begin{aligned} \frac{d}{dt} \big (m(t) + n(t)\big ) + c \langle t \rangle ^{- \frac{5\alpha }{3}} \big (m(t)+n(t)\big )&\lesssim \langle t \rangle ^{- \frac{8 \alpha }{3}+ 2e} + c n(t) \langle t \rangle ^{- \frac{5 \alpha }{3}}\\&\lesssim \langle t \rangle ^{- \frac{8 \alpha }{3}+ 2e}. \end{aligned} \end{aligned}$$

Multiply the differential inequality (5.6) by $e^{cI(t)}$ for

$$\begin{aligned} I(t) = \int _0^t \langle s \rangle ^{-\frac{5\alpha }{3}} \, \textrm{d}s \sim \langle t \rangle ^{1 - \frac{5\alpha }{3}}, \end{aligned}$$

and integrate in time to get

$$ m(t) + n(t) \lesssim e^{-cI(t)}\big (m(0) + n(0)\big ) + e^{-cI(t)} \int _0^t e^{cI(s)}\langle s \rangle ^{- \frac{8 \alpha }{3}+ 2e} \, \textrm{d}s. $$

The first term in the right-hand side is decaying exponentially fast, so we turn immediately to the second term which behaves as

$$ e^{- c \langle t \rangle ^{1 - \frac{5\alpha }{3}}} \int _0^t \langle s \rangle ^{2e - \frac{8 \alpha }{3}} e^{c \langle s \rangle ^{1 - \frac{5\alpha }{3}}} \, \textrm{d}s \sim \langle t \rangle ^{2e -\alpha }, $$

where we use the identity

5.7 $$\begin{aligned} \int _1^T e^{t^a} t^b \, \textrm{d}t \sim \int _1^{T^a} e^s s^{\frac{b}{a} + \frac{1}{a} -1} \, \textrm{d}s \sim e^{T^a} T^{b-a+1}, \qquad \text{ if } T>2, a>0, b \in \mathbb {R} \end{aligned}$$

(which is itself a consequence of $\int _1^t e^s s^a \, \textrm{d}s \sim e^t t^a$, valid for any $a \in \mathbb {R}$). Thus we conclude that indeed $m(t) \lesssim \langle t \rangle ^{2e -\alpha }.$

Before we finish the proof we briefly verify the remaining claim that the matrix $G_{\mathcal {B}_{t,\alpha }}$ is invertible for *t* sufficiently large: This claim follows immediately using the fact that the set of invertible matrices is open subset of the space of 2×2 matrices. Indeed we know that the full Gram matrix $G_{\mathbb {T}}$ is invertible since $\phi _1, \phi _2$ are linearly independent. Also, we write that

$$G_{\mathcal {B}_{t,\alpha }} = G_{\mathbb {T}} - G_{\mathcal {E}_{t,\alpha }}, \quad \text { with }\ \left( G_{\mathcal {E}_{t,\alpha }}\right) _{i,j=1}^2:= \int _{\mathcal {E}_{t,\alpha }} \phi _i \phi _j, $$

and we have

$$\left| \left( G_{\mathcal {E}_{t,\alpha }} \right) _{i,j}\right| \lesssim |\mathcal {E}_{t,\alpha }|\lesssim \langle t \rangle ^{-\alpha }, $$

since $\phi _i, \phi _j$ are bounded. Thus,

$$ G_{\mathcal {E}_{t,\alpha }} \rightarrow 0, \quad t\rightarrow \infty \quad \Rightarrow \quad G_{\mathcal {B}_{t,\alpha }} \rightarrow G_{\mathbb {T}}.$$

Since $G_{\mathbb {T}}$ is invertible and the set of invertible matrices is open, it follows that $G_{\mathcal {B}_{t,\alpha }}$ is invertible for all sufficiently large *t*. □

### A weak pointwise bound

We prove a very weak bound, which will be a stepping stone to start the proof of pointwise decay.

#### Lemma 7

Let *g* solve $\partial _t g - L g =0$ with $g(t=0) =g_0 \in L^\infty$. Then

$$ \Vert g(t) \Vert _{L^\infty (\mathbb {T})} \lesssim \langle t \rangle ^{1+} \Vert g_0 \Vert _{L^\infty (\mathbb {T})}. $$

#### Proof

By the decomposition of *L* in Section 4, *f* solves the equation

$$ \partial _t g = -ag - K_1 g + 2K_2 g. $$

We now want to bound the right-hand side in order to obtain an ODE satisfied by $\Vert g(t) \Vert _{L^\infty }$. Since a≥0,

$$ \frac{d}{dt} \Vert g \Vert _{L^\infty } \lesssim \Vert K_1 g \Vert _{L^\infty } + \Vert K_2 g \Vert _{L^\infty }. $$

The kernel of $K_2$ is $\displaystyle \frac{\omega \omega _1 \omega _2 \omega _3 \mathfrak {f}_1 \mathfrak {f}_3}{F_+(p,p_2)} \lesssim \sqrt{\omega \omega _2} \omega _1 \omega _3$; hence it is uniformly bounded and

$$ \Vert K_2 g(t) \Vert _{L^\infty } \lesssim \Vert g(t) \Vert _{L^2} \lesssim \Vert g_0\Vert _{L^\infty }, $$

since the $L^2$ norm of *f* is decreasing.

Turning to $K_1$, its kernel is $\displaystyle \frac{\omega \omega _1 \omega _2 \omega _3 \mathfrak {f}_2 \mathfrak {f}_3}{\sqrt{F_-(p,p_1)}} \textbf{1}_{F_-(p,p_1)>0}$. We learn from Lemma 15 that $F_-$ has two nontrivial zeros, $p_1'$ and $p_1''$ and that $F_-(p,\cdot )$ is only positive on $[0,p_1'] \cup [p_1'',2\pi ]$. We only consider the first interval for simplicity and we apply Lemma 19 followed by lower bound on $F_-$ in Lemma 15 to get that

$$\begin{aligned} \left| \int _0^{p_1'} \frac{\omega \omega _1 \omega _2 \omega _3 \textbf{1}_{F_->0}}{\sqrt{F_-}} g(t,p_1) \, \textrm{d}p_1 \right|&\lesssim \omega ^2 \left| \int \frac{ \textbf{1}_{F_->0}}{\sqrt{F_-}} g(t,p_1) \, \textrm{d}p_1 \right| \\&\lesssim \omega ^{\frac{3}{2}} \left| \int _0^{p_1'} \frac{1}{\sqrt{p_1'-p_1}} g(t,p_1) \, \textrm{d}p_1 \right| . \end{aligned}$$

We then split the integral between $|p-p_1'| < \langle t \rangle ^{-N}$ and $|p-p_1'| > \langle t \rangle ^{-N}$ for *N* large enough and bound the above by

$$\begin{aligned} \int _{|p_1-p_1'| > \langle t \rangle ^{-N}} \left| \frac{g(t,p_1)}{\sqrt{p_1'-p_1}} \right| \, \textrm{d}p_1 + \int _{|p_1-p_1'| < \langle t \rangle ^{-N} } \left| \frac{g(t,p_1)}{\sqrt{p_1'-p_1}} \right| \, \textrm{d}p_1. \end{aligned}$$

Bounding the first integral by the Cauchy-Schwarz inequality and the second by the $L^\infty$ norm of *g*, this is less than

$$ \log \langle t \rangle \Vert g (t)\Vert _{L^2} + \langle t \rangle ^{-\frac{N}{2}} \Vert g(t) \Vert _{L^\infty } \lesssim \log \langle t \rangle \Vert g_0\Vert _{L^2} + \langle t \rangle ^{-\frac{N}{2}} \Vert g(t) \Vert _{L^\infty }, $$

since the $L^2$ norm of *g* is decreasing.

Overall, we find the ODE

$$ \frac{d}{dt} \Vert g(t) \Vert _{L^\infty } \lesssim \log \langle t \rangle \Vert g_0\Vert _{L^2} + \langle t \rangle ^{- \frac{N}{2}} \Vert g(t) \Vert _{L^\infty }, $$

which gives the desired result upon integration. □

### Pointwise bounds

#### Lemma 8

Let $\alpha < \frac{3}{5}$, and assume that

$$ \Vert g_0 \Vert _{L^\infty } \le 1 \quad \text{ and } \quad \Vert g(t) \Vert _{L^2} \le \langle t \rangle ^b. $$

(i) If b>-1, for any $p \in [0,2\pi ]$,
  $$\begin{aligned} |g(t,p)| \lesssim |g_0(p)| + \omega ^{\frac{3}{2}-} \langle t \rangle ^{b+1+}. \end{aligned}$$
(ii) In the bulk: if $\omega > \langle t \rangle ^{-\alpha }$,
  $$\begin{aligned} |g(t,p)| \lesssim \omega ^{-\frac{1}{6}} \langle t \rangle ^{b+} \end{aligned}$$

#### Proof

(*i*) We first split as above into the two integral kernels

$$\begin{aligned} \frac{d}{dt} |g(p)|&\lesssim \left| \int \frac{\omega \omega _1 \omega _2 \omega _3}{\sqrt{F_+}} g(p_2) \, \textrm{d}p_2 \right| + \left| \int \frac{\omega \omega _1 \omega _2 \omega _3 1_{F_- >0}}{\sqrt{F_-}} g(p_1) \, \textrm{d}p_1 \right| = I_+ + I_-. \end{aligned}$$

We start with $I_+$, for which we will prove that when *p* is close to 0,

$$\begin{aligned} I_+ \lesssim \omega ^{\frac{3}{2}}\left( 1+ \sqrt{ \log \frac{2}{\omega }}\right) \Vert g\Vert _{L^2(\mathbb {T})}. \end{aligned}$$

The case *p* close to 2π is symmetric, and if *p* is away from the edges, the integral $I_+$ is easy since then $F_+$ is lower bounded. Indeed one checks that the only zeros of $F_+$ are the points (0,2π) and (2π,0).

Thus we consider $0<\theta \ll 1$ so that $p\in [0,\theta ]$ and, following the notation of Lemmas 18, 19, we set $\varepsilon : = \sin \frac{p}{2}$ and, $s:=\sin \frac{2\pi - p_2}{2}$.

We split the $p_2$ integral into two domains close and further away from 2π, namely $[0, 2\pi - c_0]$ and $[2\pi - c_0, 2\pi ]$ for $c_0>0$ is a small constant. Then:

$$\begin{aligned}  &   \left| \int \frac{\omega \omega _1 \omega _2 \omega _3}{\sqrt{F_+}} g(p_2) \, \textrm{d}p_2 \right| \lesssim \left| \int _{0}^{2\pi - c_0} \frac{\omega \omega _1 \omega _2 \omega _3}{\sqrt{F_+}} g(p_2) \, \textrm{d}p_2\right| \\  &   + \left| \int _{2\pi - c_0}^{2\pi } \frac{\omega \omega _1 \omega _2 \omega _3}{\sqrt{F_+}} g(p_2) \, \textrm{d}p_2 \right| := I_+^{b}(p) + I_+^{e}(p). \end{aligned}$$

For $I_+^b(p)$: We notice that $F_+$ is lower bounded since $p_2$ stays away from 2π in that region, and $p \in [0,\theta ]$ with θ small (so $F_+$ has no zeros). From Lemma 19 we have that $\omega _1\omega _2\omega _3 \lesssim \omega = \varepsilon$. Thus the integral kernel is bounded by $\lesssim \varepsilon ^2$. Cauchy-Schwarz then yields

$$\begin{aligned} I_+^b \lesssim \varepsilon ^2 \Vert g\Vert _{L^2}. \end{aligned}$$

For $I_+^e(p)$: we notice that when $p_2 \in (2\pi - c_0, 2\pi )$, $s\le s_0$ for some $s_0 \sim c_0$. From the proof of Lemma 18 we learn that $\omega _1\omega _2\omega _3 \sim \frac{\varepsilon s^2}{\varepsilon ^2 + s^2}$ and also that

$$\begin{aligned} F_+ (p,p_2) = \frac{(\varepsilon ^2 - s^2)^2}{\big (\sqrt{1-\varepsilon ^2} + \sqrt{1-s^2} \big )^2} + 4 \varepsilon s. \end{aligned}$$

The denominator is lower and upper bounded and so $F_+ (p,p_2) \sim (\varepsilon ^2 - s^2)^2 +4 \varepsilon s$. Thus,

$$\begin{aligned} I_+^e(p) \lesssim \left| \int _{2\pi - c_0}^{2\pi } \frac{\varepsilon ^2\,s^2}{\varepsilon ^2 + s^2} \frac{1}{\sqrt{(\varepsilon ^2 - s^2)^2 +4 \varepsilon s}} g(p_2) \, \textrm{d}p_2 \right| . \end{aligned}$$

Now if $0 < s \le \varepsilon$, we have $\varepsilon ^2 + s^2 \sim \varepsilon ^2$ and the denominator is lower bounded by $\sqrt{\varepsilon s}$. The integral kernel then is $\lesssim \frac{s^2}{\sqrt{\varepsilon s}} = s^{\frac{3}{2}}\varepsilon ^{-\frac{1}{2}} \lesssim \varepsilon$. In that case, by Cauchy-Schwarz,

$$\begin{aligned} \int _{0<s\le \varepsilon }|g(p_2)| \varepsilon \, \textrm{d}p_2 \le \varepsilon ^{\frac{3}{2}} \Vert g\Vert _{L^2}. \end{aligned}$$

Finally if $\varepsilon < s \le s_0$, we have $\varepsilon ^2 + s^2 \sim s^2$ and the denominator is again lower bounded by $\sqrt{\varepsilon s}$. The integral kernel then is $\lesssim \frac{\varepsilon ^2}{\sqrt{\varepsilon s}} = \varepsilon ^{\frac{3}{2}}s^{-\frac{1}{2}}.$ Then, for any $\kappa _0>0$,

$$\begin{aligned} \int _{\varepsilon \le s \le s_0} |g(p_2)|\varepsilon ^{\frac{3}{2}}s^{-\frac{1}{2}} \, \textrm{d}p_2 \lesssim \varepsilon ^{\frac{3}{2}} \left( \int _{\varepsilon }^{s_0} \frac{1}{s} \right) ^{1/2} \Vert g\Vert _{L^2} \lesssim \varepsilon ^{\frac{3}{2}} \sqrt{\log \left( \frac{s_0}{\varepsilon } \right) }\Vert g\Vert _{L^2} \lesssim _{\kappa _0} \omega ^{\frac{3}{2} - \kappa _0} \langle t \rangle ^b. \end{aligned}$$

Altogether,

$$I_+ = I_+^b+ I_+^e(p)\lesssim _{\kappa _0} \omega ^2\langle t \rangle ^b + \omega ^{\frac{3}{2} - \kappa _0}\langle t \rangle ^b \lesssim _{\kappa _0} \omega ^{\frac{3}{2} - \kappa _0 }\langle t \rangle ^b.$$

For the singular term $I_-$, we first use Lemma 19 to obtain that

$$ I_- \le \left| \int \frac{\omega \omega _1 \omega _2 \omega _3 \textbf{1}_{F_->0}}{\sqrt{F_-}} g(p_1) \, \textrm{d}p_1 \right| \lesssim \omega \left| \int \frac{\omega \textbf{1}_{F_->0}}{\sqrt{F_-}} g(p_1) \, \textrm{d}p_1 \right| . $$

Next, we resort to Lemma 15, from which we learn that $F_-(p,\cdot )$ is positive in $[0,p_2'] \cup [p_2'',2\pi ]$; for simplicity, we will focus on the left interval. Using the lower bound on $F_-$ in Lemma 15, we obtain that

$$ \omega \left| \int _0^{p_2'} \frac{\omega \textbf{1}_{F_->0}}{\sqrt{F_-}} g(p_2) \, \textrm{d}p_2 \right| \lesssim \omega ^{\frac{3}{2}} \left| \int _0^{p_2'} \frac{1}{\sqrt{p_2'-p_2}} g(p_2) \, \textrm{d}p_2 \right| $$

Finally, splitting the integral between $|p-p_1'| < \langle t \rangle ^{-N}$ and $|p-p_1'| > \langle t \rangle ^{-N}$ for *N* big enough, and using Lemma 7, we get

$$ I_- \lesssim \omega ^{\frac{3}{2}} \left[ \log \langle t \rangle \Vert g \Vert _{L^2} + \langle t \rangle ^{-\frac{N}{2}} \Vert g \Vert _{L^\infty } \right] \lesssim \omega ^{\frac{3}{2}} \langle t \rangle ^{b+} + \omega ^{\frac{3}{2}} \langle t \rangle ^{2-\frac{N}{2}}. $$

We now combine the estimates on $I_+$ and $I_-$ to integrate the previous ODE in time, which gives

$$ |g(t,p)| \lesssim | g_{0}(p) |+ \omega ^{\frac{3}{2}} \langle t \rangle ^{ b + 1+}. $$

(*ii*) We proceed as in (*i*), except that we do not neglect the term *a*(*p*)*g*(*t*, *p*). This gives the differential inequality

$$ \left| \frac{d}{dt} g(t,p) + a(p) g(t,p) \right| \lesssim \omega ^{\frac{3}{2}} \langle t \rangle ^{b+} $$

or

$$ \left| \frac{d}{dt} \left[ e^{a(p) t} g(t,p) \right] \right| \lesssim e^{a(p) t} \omega ^{\frac{3}{2}} \langle t \rangle ^{b+}, $$

which can be integrated to give

$$ |g(t,p)| \lesssim e^{- a(p) t} g_0(p) + \omega ^{\frac{3}{2}} e^{-a(p) t} \int _0^t e^{a(p)s} \langle s \rangle ^{b+} \, \textrm{d}s. $$

If $\omega > \langle t \rangle ^{-\alpha }$, the first term on the right-hand side decays faster than any polynomial. Turning to the second term, we bound it in a straightforward way and use that $a(p) \sim \omega ^{5/3}$ to get

$$ \left| \omega ^{\frac{3}{2}} e^{-a(p) t} \int _0^t e^{a(p)s} \langle s \rangle ^{b+} \, \textrm{d}s \right| \lesssim \omega ^{\frac{3}{2}} \langle t \rangle ^{b+} e^{-a(p) t} \int _0^t e^{a(p)s} \, \textrm{d}s \lesssim \omega ^{- \frac{1}{6}} \langle t \rangle ^{b+}. $$

□

### Iterative improvement

We will now apply iteratively Lemmas 6 and 8, having set $\alpha = \frac{3}{5} - \epsilon _0$, with $0 <\epsilon _0 \ll 1$. In the following, we denote $t^{a +}$ for $t^{a + C\epsilon _0}$, for a constant *C*, instead of our usual notation $t^{a+}$ meaning $t^{a + \delta }$ for any δ>0.

- applying First Lemma 8 (*i*) with b=0 gives, provided that $g_0 \in L^2 \cap \big ( \operatorname {Ker} L \big )^\perp$,
  $$ |g(t,p)| \lesssim |g_0(p)| + \omega ^{\frac{3}{2}} \langle t \rangle ^{1+}. $$
  Thus, assuming $|g_0(p)| \le 1$, we get
  $$ q(t) \lesssim 1 + \langle t \rangle ^{1 - \frac{3}{2} \cdot \frac{3}{5} +} \lesssim \langle t \rangle ^{ \frac{1}{10} +}. $$
- Applying Lemma 6 with $e = \frac{1}{10} +$ gives
  $$ m(t) \lesssim \langle t \rangle ^{\frac{1}{5} - \frac{3}{5} +} = \langle t \rangle ^{- \frac{2}{5} +}. $$
  This gives
  $$ \Vert g(t) \Vert _{L^2} \lesssim m(t)^{\frac{1}{2}} + q(t) \langle t \rangle ^{-\frac{3}{10} +} \sim \langle t \rangle ^{-\frac{1}{5} +}. $$
- Applying Lemma 8 (*i*) with $b = - \frac{1}{5} +$ gives
  $$ |g(t,p)| \lesssim |g_0(p)| + \omega ^{\frac{3}{2}} \langle t \rangle ^{\frac{4}{5}+}. $$
  Assuming $|g_0(p)| \lesssim \omega ^{\frac{1}{6}}$, this gives
  $$ q(t) \lesssim \langle t \rangle ^{- \frac{1}{6} \cdot \frac{3}{5} +} + \langle t \rangle ^{\frac{4}{5} - \frac{3}{2} \cdot \frac{3}{5} +} \sim \langle t \rangle ^{- \frac{1}{10} +}. $$
- Applying Lemma 6 with $e = - \frac{1}{10}$ gives
  $$ m(t) \lesssim \langle t \rangle ^{-\frac{1}{5} - \frac{3}{5} +} = \langle t \rangle ^{-\frac{4}{5} +}, $$
  hence
  $$ \Vert g(t) \Vert _{L^2} \lesssim \langle t \rangle ^{-\frac{2}{5} +} + \langle t \rangle ^{-\frac{1}{10} - \frac{3}{10} +} \sim \langle t \rangle ^{-\frac{2}{5} +}. $$
- Applying Lemma 8 (*i*) with $b = - \frac{2}{5}$, we get
  $$ |g(t,p)| \lesssim |g_0(p)| + \omega ^{\frac{3}{2} } \langle t \rangle ^{\frac{3}{5}+}. $$
  If $|g_0(p)| \le \omega ^\nu$ with $\nu \in [\frac{1}{6},\frac{1}{2}]$, this gives
  5.8 $$\begin{aligned} q(t) \lesssim \langle t \rangle ^{- \frac{3}{5} \nu +} + \langle t \rangle ^{- \frac{3}{10} +} \sim \langle t \rangle ^{- \frac{3}{5} \nu +}. \end{aligned}$$
- Finally, applying Lemma 6 with $e =- \frac{3}{5} \nu +$ gives
  $$ m(t) \lesssim \langle t \rangle ^{- \frac{6 \nu }{5} - \frac{3}{5} +}, $$
  and hence
  5.9 $$\begin{aligned} \Vert g(t) \Vert _{L^2} \lesssim \langle t \rangle ^{- \frac{3 \nu }{5} - \frac{3}{10} +}. \end{aligned}$$

We can now prove our final pointwise bound, under the assumption made above that $|g_0(p)| \le \omega ^\nu$. In the edges, we use (5.8) to get that

$$ \omega ^\mu | g(t,p) | \lesssim \langle t \rangle ^{-\frac{3}{5} (\mu + \nu ) +} \qquad \text{ if } \omega < \langle t \rangle ^{-\alpha }. $$

In the bulk, we use Lemma 8 (*ii*) and (5.9) to get that

$$ \omega ^{\frac{1}{6}} | g(t,p) | \lesssim \langle t \rangle ^{- \frac{6 \nu + 3}{10}} \qquad \text{ if } \omega > \langle t \rangle ^{-\alpha }. $$

Combining these two bounds results in the following theorem:

#### Theorem 3

(Pointwise decay) Assume that $g_0 \in L^\infty$, with a zero projection (in $L^2$) on $\operatorname {Ker} L$. Then for any $\mu ,\nu \in (\frac{1}{6}, \frac{1}{2})$ and any δ>0,

$$ \Vert \omega ^\mu e^{tL} g_0 \Vert _{L^\infty } \lesssim _\delta \langle t \rangle ^{- \frac{3}{5}(\mu + \nu )+ \delta } \Vert \omega ^{-\nu } g_0(p) \Vert _{L^\infty }. $$

## Nonlinear stability

### Theorem 4

For any $\beta ,\gamma >0$, there exists $\epsilon _0$ such that the following holds. if

$$ f(t=0) = \mathfrak {f}_{\beta ,\gamma } [ 1 + g_0] $$

where

$$ \int \mathfrak {f}_{\beta ,\gamma } g_0 \, \textrm{d}p = \int \omega \mathfrak {f}_{\beta ,\gamma } g_0 \, \textrm{d}p = 0 $$

and

$$ \Vert \omega ^{-\frac{1}{2}} g_0 \Vert _{L^\infty } = \epsilon < \epsilon _0, $$

then there exists a global solution which can be written as

$$ f(t) = \mathfrak {f}_{\beta ,\gamma } [ 1 + g] \qquad \text{ where } \quad \Vert \omega ^{\frac{1}{2}} g(t,p) \Vert _{L^\infty _p} \lesssim \epsilon \langle t \rangle ^{-\frac{3}{5} + \frac{1}{1000}} \quad \text{ for } \text{ all } t \ge 0. $$

### Proof

The full equation satisfied by the perturbation *g* is

6.1 $$\begin{aligned} \partial _t g - L g = \mathcal {Q}(g) + \mathcal {C}(g), \end{aligned}$$

where

$$\begin{aligned}&\mathcal {Q}[g](t,p_0)= \frac{2}{\mathfrak {f}_0} \int _0^{2\pi } \frac{ \omega _0 \omega _1 \omega _2 \omega _3 }{\sqrt{F_+(p_0,p_2)}} \left[ \mathfrak {f}_2 \mathfrak {f}_3 g_2 g_3 - \mathfrak {f}_0 \mathfrak {f}_1 g_0 g_1 \right] \, \textrm{d}p_2 \\&\mathcal {C}[g](t,p_0)= \frac{1}{\mathfrak {f}_0} \int _0^{2\pi } \frac{ \omega _0 \omega _1 \omega _2 \omega _3 }{\sqrt{F_+(p_0,p_2)}} \prod _{\ell =0}^3 \mathfrak {f}_\ell g_\ell \left( \frac{1}{\mathfrak {f} g}+\frac{1}{\mathfrak {f}_1 g_1}-\frac{1}{\mathfrak {f}_2 g_2}-\frac{1}{\mathfrak {f}_3 g_3}\right) \, \textrm{d}p_2. \end{aligned}$$

Duhamel’s formula gives the equivalent formulation:

6.2 $$\begin{aligned} g(t) = S_t g_0 + \int _0^t S_{t-s} \left( \mathcal {Q}[g](s,p) + \mathcal {C}[g](s,p)\right) \, \textrm{d}s. \end{aligned}$$

The key norm that will be used to analyze this problem is, for T>0,

6.3 $$\begin{aligned} \Vert g \Vert _{\mathcal {B}_T} := \left\| \langle t \rangle ^{\frac{2}{5} - \delta } \omega ^{\frac{1}{6}} g \right\| _{L^\infty _{t,p}([0,T] \times \mathbb {T})} + \left\| \langle t \rangle ^{\frac{3}{5} - \delta } \omega ^{\frac{1}{2}} g \right\| _{L^\infty _{t,p}([0,T] \times \mathbb {T})}. \end{aligned}$$

Our first lemma gives local well-posedness in $\omega ^{- \frac{1}{6}} L^\infty$ for this equation.

### Lemma 9

(Local well-posedness in $\omega ^{-\frac{1}{6}} L^\infty$) The equation (6.1) admits a unique maximal solution $g \in \mathcal {C}([0,T_0), \omega ^{-\frac{1}{6}} L^\infty )$, where $T_0 >0$ and

$$ \lim _{t \rightarrow T_0} \Vert \omega ^{\frac{1}{6}} g(t,p) \Vert _{L^\infty _p} = \infty \qquad \text{ if } T_0 < \infty . $$

Furthermore, there exists a constant $C_0$ such that

$$ \lim _{t \rightarrow 0} \Vert g \Vert _{\mathcal {B}_t} \le C_0 \epsilon , $$

where $\epsilon := \Vert \omega ^{-\frac{1}{2}} g_0 \Vert _{L^\infty }$ and where the norm $\Vert \cdot \Vert _{\mathcal {B}_T}$ is defined in (6.3).

We postpone the proof of this lemma for the time being, and admit its statement. We aim at proving that $T_0 = \infty$, and that the solution *g* is actually decaying. This will be achieved through a boostrap argument bearing on the quantity $\Vert f \Vert _{\mathcal {B}_t}$. This bootstrap argument will rely on the two lemmas, whose proofs we postpone for the moment.

### Lemma 10

(A priori bound) There exists a constant $C_0 >0$ such that, for any T>0, if *g* is a solution in $\mathcal {C}([0,T],\omega ^{- \frac{1}{6}} L^\infty )$, then

$$ \Vert g \Vert _{\mathcal {B}_T} \le C_0 \left[ \epsilon + \Vert g \Vert _{\mathcal {B}_T}^2 + \Vert g \Vert _{\mathcal {B}_T}^3 \right] , $$

where $\epsilon := \Vert \omega ^{-\frac{1}{2}} g_0 \Vert _{L^\infty }$ and where $\Vert \cdot \Vert _{\mathcal {B}_T}$ is defined in (6.3).

In the above lemma, we denoted $C_0$ for the constant, just like in Lemma 9; it suffices to take $C_0$ to be the largest of the two to avoid any confusion.

### Lemma 11

(Bootstrap inequality) If *x*(*t*) is a continuous function on (0, *T*) such that

$$ x(t) \le C_0 \left[ \epsilon + x(t)^2 + x(t)^3 \right] \qquad \text{ and } \qquad \lim _{t\rightarrow 0} x(t) \le C_0 \epsilon , $$

then

$$ x(t) \le 2C_0 \epsilon \qquad \text{ for } \text{ any } \; t \in (0,T). $$

We now perform the bootstrap argument. Arguing by contradiction, let us assume that $T_0 < \infty$. By Lemmas 10 and 11, we get that $\Vert g \Vert _{\mathcal {B}_t} \le 2 C_o \epsilon$ for all $t < T_0$. There this contradicts the blow up criterion in Lemma 9. Therefore, $T_0 = \infty$. A new application of Lemmas 10 and 11 gives the desired result! □

### Proof of Lemma 9

It is very similar to the proof of Theorem 1 above and Lemma 10 below, and will therefore be omitted. □

### Proof of Lemma 10

The linear term on the right-hand side of (6.2) can be bounded by Theorem 3:

$$ \left\| S_t g_0 \right\| _{\mathcal {B}_\infty } \lesssim \Vert \omega ^{-\frac{1}{2}} g \Vert _{L^\infty } < \epsilon . $$

To deal with the quadratic and cubic terms, we will use the conservation laws of mass and energy. These imply that

$$\begin{aligned}&\int \mathfrak {f}_{\beta ,\gamma }(p)(1 + g(t,p)) \, \textrm{d}p = \int \mathfrak {f}_{\beta ,\gamma }(p) \, \textrm{d}p \\&\int \omega (p) \mathfrak {f}_{\beta ,\gamma }(p)(1 + g(t,p)) \, \textrm{d}p = \int \omega (p) \mathfrak {f}_{\beta ,\gamma }(p) \, \textrm{d}p, \end{aligned}$$

or, in other words,

$$ \int \mathfrak {f}_{\beta ,\gamma }(p) g(t,p) \, \textrm{d}p = \int \omega (p) \mathfrak {f}_{\beta ,\gamma }(p) g(t,p) \, \textrm{d}p = 0. $$

As a consequence, the orthogonal projection (in $L^2$) of *g* and *Lg* on $\operatorname {Ker} L$ is zero. For the equation (6.2) satisfied by *g*, this implies that the projection of the quadratic and cubic terms on $\operatorname {Ker} L$ is also zero. Therefore, we can apply Theorem 3 with $\mu = \nu = \frac{1}{2}$: if t∈[0,T],

$$\begin{aligned}&\left\| \omega ^{\frac{1}{2}} \int _0^t S_{t-s} \left( \mathcal {Q}[g](s,p) + \mathcal {C}[g](s,p)\right) \, \textrm{d}s\right\| _{L^\infty _p} \\&\qquad \lesssim \int _0^t \langle t-s \rangle ^{-\frac{3}{5} + \delta } \left\| \omega ^{-\frac{1}{2}} \mathcal {Q}[g](s,p)\right\| _{L^\infty } \, \textrm{d}s + \int _0^t \langle t-s \rangle ^{-\frac{3}{5} + \delta } \left\| \omega ^{-\frac{1}{2}} \mathcal {C}[g](s,p) \right\| _{L^\infty } \, \textrm{d}s\\&\qquad = I + II. \end{aligned}$$

Using that $F_+ \gtrsim \omega _0 \omega _2$ and $\omega _1 \omega _2 \omega _3 \lesssim \omega _0$,

$$\begin{aligned} I&\lesssim \int _0^t \langle t-s \rangle ^{-\frac{3}{5} + \delta }\left\| \int \sqrt{\omega _2} \omega _1 \omega _3 [ |g_2 g_3| + |g_0 g_1|] \, \textrm{d}p_2 \right\| _{L^\infty } \, \textrm{d}s \\&\lesssim \int _0^t \langle t-s \rangle ^{-\frac{3}{5} + \delta } \Vert \omega ^{\frac{1}{2}}g \Vert _{L^\infty }^2 \, \textrm{d}s \lesssim \Vert g\Vert _{\mathcal {B}}^2 \int _0^t \langle t-s \rangle ^{-\frac{3}{5} + \delta } \langle s \rangle ^{\left( - \frac{3}{5} + \delta \right) \cdot 2} \, \textrm{d}s \\&\lesssim \Vert g \Vert _{\mathcal {B}}^2 \langle t \rangle ^{- \frac{3}{5} + \delta }. \end{aligned}$$

Similarly,

$$\begin{aligned} II&\lesssim \int _0^t \langle t-s \rangle ^{-\frac{3}{5} + \delta }\left\| \int \sqrt{\omega _2} \omega _1 \omega _3 [ |g_1 g_2 g_3| + |g_0 g_2 g_3| + |g_0 g_1 g_2| + |g_0 g_1 g_3| ] \, \textrm{d}p_2 \right\| _{L^\infty } \, \textrm{d}s \\&\lesssim \int _0^t \langle t-s \rangle ^{-\frac{3}{5} + \delta } \Vert \omega ^{\frac{1}{6}} g \Vert _{L^\infty } \Vert \omega ^{\frac{1}{3}} g \Vert _{L^\infty } \Vert \omega ^{\frac{1}{2}} g \Vert _{L^\infty } \, \textrm{d}s \\&\lesssim \Vert g \Vert _{\mathcal {B}}^3 \int _0^t \langle t-s \rangle ^{-\frac{3}{5} + \delta } \langle s \rangle ^{ -\frac{2}{5} - \frac{1}{2} - \frac{3}{5} + 4\delta } \, \textrm{d}s \\&\lesssim \Vert g\Vert _{\mathcal {B}}^3 \langle t \rangle ^{-\frac{3}{5} + \delta }. \end{aligned}$$

Using Theorem 3 with $\mu = \frac{1}{6}$ and $\nu = \frac{1}{2}$,

$$\begin{aligned}&\left\| \omega ^{\frac{1}{6}} \int _0^t S_{t-s} \left( \mathcal {Q}[g](s,p) + \mathcal {C}[g](s,p)\right) \, \textrm{d}s\right\| _{L^\infty _p} \\&\qquad \lesssim \int _0^t \langle t-s \rangle ^{-\frac{2}{5} + \delta } \left\| \omega ^{-\frac{1}{2}} \mathcal {Q}[g](s,p)\right\| _{L^\infty } \, \textrm{d}s + \int _0^t \langle t-s \rangle ^{-\frac{2}{5} + \delta } \left\| \omega ^{-\frac{1}{2}} \mathcal {C}[g](s,p) \right\| _{L^\infty } \, \textrm{d}s\\&\qquad = III + IV. \end{aligned}$$

Taking of advantage the inequalities $F_+ \gtrsim \omega _0 \omega _2$ and $\omega _1 \omega _2 \omega _3 \lesssim \omega _0$,

$$\begin{aligned} III&\lesssim \int _0^t \langle t-s \rangle ^{-\frac{2}{5} + \delta }\left\| \int \sqrt{\omega _2} \omega _1 \omega _3 [ |g_2 g_3| + |g_0 g_1|] \, \textrm{d}p_2 \right\| _{L^\infty } \, \textrm{d}s \\&\lesssim \int _0^t \langle t-s \rangle ^{-\frac{2}{5} + \delta } \Vert \omega ^{\frac{1}{2}} g \Vert _{L^\infty _p} \, \textrm{d}s \\&\lesssim \Vert g \Vert _{\mathcal {B}_t}^2 \int _0^t \langle t-s \rangle ^{-\frac{2}{5} + \delta } \langle s \rangle ^{\left( \frac{1}{2} + \delta \right) \cdot 2} \\&\lesssim \Vert g \Vert _{\mathcal {B}_t}^2 \langle t \rangle ^{- \frac{2}{5} + \delta }. \end{aligned}$$

Similarly,

$$\begin{aligned} IV&\lesssim \int _0^t \langle t-s \rangle ^{-\frac{2}{5} + \delta }\left\| \int \sqrt{\omega _2} \omega _1 \omega _3 [ |g_1 g_2 g_3| + |g_0 g_2 g_3| + |g_0 g_1 g_2| + |g_0 g_1 g_3| ] \, \textrm{d}p_2 \right\| _{L^\infty } \, \textrm{d}s \\&\lesssim \int _0^t \langle t-s \rangle ^{-\frac{2}{5} + \delta } \Vert \omega ^{\frac{1}{6}} g \Vert _{L^\infty } \Vert \omega ^{\frac{1}{3}} g \Vert _{L^\infty } \Vert \omega ^{\frac{1}{2}} g \Vert _{L^\infty } \, \textrm{d}s \\&\lesssim \Vert g \Vert _{\mathcal {B}}^3 \int _0^t \langle t-s \rangle ^{-\frac{2}{5} + \delta } \langle s \rangle ^{ -\frac{2}{5} - \frac{1}{2} - \frac{3}{5} + 4\delta } \, \textrm{d}s \\&\lesssim \Vert g \Vert _{\mathcal {B}}^3 \langle t \rangle ^{-\frac{2}{5} + \delta }. \end{aligned}$$

This gives the desired estimate. □

### Proof of Lemma 11

Consider the function

$$ F: x \mapsto x - C_0 (\epsilon + x^2 + x^3). $$

It is clear that *F* is negative on $[0,C_0 \epsilon + \eta ]$, for some η>0. It is also clear that

$$ F(2C_0 \epsilon ) = C_0 \epsilon - 4 C_0^3 \epsilon ^2 - 8 C_0^4 \epsilon ^3 = C_0 \epsilon (1 - 4 C_0^2 \epsilon - 8 C_0^3 \epsilon ^2) \ge C_0 \epsilon (1 - 4 C_0^2 \epsilon _0 - 8 C_0^3 \epsilon _0^2) \ge 0, $$

provided that $\epsilon _0$ is chosen sufficiently small. The statement of the lemma follows by the intermediate value theorem. □

## Mass and Energy of RJ spectra

In this section, we investigate the following question: given a mass and an energy $(\mathcal {M}_0, \mathcal {E}_0)$, is there a Rayleigh–Jeans spectrum $\mathfrak {f}$ such that $\mathcal {M}(\mathfrak {f}) = \mathcal {M}_0, \mathcal {E}(\mathfrak {f}) = \mathcal {E}_0$? It turns out that the ratio $\mathcal {E}_0/\mathcal {M}_0$ decides whether such a RJ spectrum exists. We define the following functions:

$$\begin{aligned} \begin{aligned} \mathcal {M} (b, g)&= \int _{\mathbb {T}} \frac{\, \textrm{d}p}{b^{-1} \omega + g^{-1}}, \\ \mathcal {E} (b,g)&=\int _{\mathbb {T}} \frac{\omega \, \textrm{d}p}{b^{-1} \omega + g^{-1}}. \end{aligned} \end{aligned}$$

Here 0<b,g<∞. We readily see that $0< \mathcal {M}(b, g), \mathcal {E} (b,g) < \infty ,$ and

$$\begin{aligned} b^{-1} \mathcal {E} + g^{-1} \mathcal {M} = 2\pi . \end{aligned}$$

Moreover, we have the following differential equation:

$$\begin{aligned} b \partial _b \mathcal {M} + g \partial _g \mathcal {M} = \mathcal {M}. \end{aligned}$$

We adopt polar coordinates to represent (*b*, *g*):

$$\begin{aligned} b = r \cos \theta , \qquad g = r \sin \theta . \end{aligned}$$

Here 0<r<∞ and $0< \theta < \pi /2$. By homogeneity,

$$\begin{aligned} \mathcal {M} (b, g) = r \mathcal {M} (\cos \theta , \sin \theta ). \end{aligned}$$

Therefore, for a given pair of positive numbers $(\mathcal {M}_0, \mathcal {E}_0)$, there exists (*b*, *g*) such that $(\mathcal {M}_0, \mathcal {E}_0) = (\mathcal {M}(b,g), \mathcal {E}(b,g))$ if and only if either of the following equations on θ are solvable:

$$\begin{aligned} \frac{\mathcal {E}_0}{\cos \theta } + \frac{\mathcal {M}_0}{\sin \theta } = 2\pi r = \frac{2\pi \mathcal {M}_0}{\mathcal {M}(\cos \theta , \sin \theta )}, \end{aligned}$$

or

$$\begin{aligned} \frac{\mathcal {E}_0}{\mathcal {M}_0} = \frac{2\pi \cos \theta }{\mathcal {M}(\cos \theta , \sin \theta )} - \cot \theta = \frac{1}{\frac{1}{2\pi } \int _{\mathbb {T}} \frac{\, \textrm{d}p}{\omega +\cot \theta }} - \cot \theta . \end{aligned}$$

Since $\theta \rightarrow \cot \theta$ is a bijection from (0,π/2) to (0,∞), this is equivalent to the fact that

$$\begin{aligned} \frac{\mathcal {E}_0}{\mathcal {M}_0} \in F((0, \infty )), \end{aligned}$$

where

$$F(\ell ) = \frac{1}{\frac{1}{2\pi } \int _{\mathbb {T} } \frac{\, \textrm{d}p}{\omega + \ell }} - \ell .$$

Note that

$$ F(0)=0 \quad \text{ while } \quad F(\infty )=\frac{2}{\pi }. $$

To see the value for F(∞), we expand for ℓ large, we write that $\frac{1}{\omega + \ell } = \frac{1}{\ell } - \frac{\omega }{\ell ^{2}} + O (\ell ^{-3})$, and thus

$$\frac{1}{2\pi } \int _{\mathbb {T}} \frac{dp}{\omega + \ell } = \frac{1}{\ell } - \frac{1}{2\pi \ell ^2} \int _{\mathbb {T}} \omega (p) dp + O (\ell ^{-3}).$$

Taking the inverse,

$$\left( \frac{1}{2\pi } \int _{\mathbb {T}} \frac{dp}{\omega + \ell } \right) ^{-1} = \ell + \frac{1}{2\pi } \int _{\mathbb {T}} \omega (p) dp + o(1)\ \Longrightarrow \ F(\infty ) = \frac{1}{2\pi } \int _{\mathbb {T}} \omega (p) dp - \frac{2}{\pi }. $$

If we let $G(\ell ) = F(\ell ) + \ell ,$ we see that

$$\frac{\, \textrm{d}}{\, \textrm{d}\ell } G(\ell ) = \frac{\frac{1}{2\pi } \int _{\mathbb {T}} \frac{1}{(\omega +\ell )^2} \, \textrm{d}p }{\left( \frac{1}{2\pi } \int _{\mathbb {T} } \frac{\, \textrm{d}p}{\omega + \ell }\right) ^2 } > 1,$$

by Jensen’s inequality (since neither $x \rightarrow x^2$ is affine nor $p \rightarrow \frac{1}{\omega +\ell }$ is constant, the inequality is strict), which implies that $\ell \rightarrow F(\ell )$ is strictly increasing. To summarize, we have the following:

### Proposition 7.1

Let $(\mathcal {M}_0, \mathcal {E}_0)$ be a pair of positive numbers. Then there exists a Rayleigh–Jeans spectrum $\mathfrak {f} = \frac{1}{\beta \omega + \gamma }$, $0< \beta , \gamma < \infty$, such that $\mathcal {M} (\mathfrak {f} ) = \mathcal {M}_0$ and $\mathcal {E} (\mathfrak {f} ) = \mathcal {E}_0$ hold if and only if

$$ 0< \frac{\mathcal {E}_0}{\mathcal {M}_0} < \frac{2}{\pi }.$$

Moreover, if the latter is the case, such $\mathfrak {f}$ is unique and determined by the following:

$$\begin{aligned} \begin{aligned} \beta&= \left( r \cos \theta \right) ^{-1}, \qquad \gamma = \left( r \sin \theta \right) ^{-1}, \\ \theta&= \arctan \left[ \left( F^{-1} \left( \frac{\mathcal {E}_0}{\mathcal {M}_0}\right) \right) ^{-1} \right] , \qquad r = \frac{ \mathcal {M}_0}{\frac{\cos \theta }{2\pi } \int _{\mathbb {T}} \frac{\, \textrm{d}p}{\omega + \cot \theta }}. \end{aligned} \end{aligned}$$

Before finishing this section, let us comment on a related conjecture made in [18]. For the truncated Wave Kinetic Equation arising from NLS, the presence or absence of a finite-time blow-up is expected to depend on whether the ratio of mass to energy is sufficiently large. Thus, given our proposition above, we expect that initial data whose mass and energy do not correspond to a Rayleigh–Jeans distribution will form a condensate in finite time. Proving rigorously that these condensates are stationary solutions remains an interesting open problem.

## Data Availability

Data sharing is not applicable to this article as no data sets were generated or analysed during the current study.

## Appendix A: Calculus lemmas

In this section, we gather some nontrivial computations which are used in the rest of the article. Many of the formulas we derive already appeared in [11] and some are new. Many proofs are also close to [11] and are provided for completeness and for the reader’s convenience.

### A.1: Integration on the resonant manifold

If $$x,y,z \in [0,2\pi ]$$, let

$$ \Omega (x,y,z) = \sin \left( \frac{x}{2} \right) + \sin \left( \frac{y}{2} \right) - \sin \left( \frac{z}{2} \right) - \left| \sin \left( \frac{x+y-z}{2} \right) \right| . $$

Recall that

$$ h(x,z) = \frac{z-x}{2} + 2 \arcsin \left( \tan \frac{|z-x|}{4} \cos \frac{z+x}{4} \right) . $$

#### Lemma 12

(Parameterization of the resonant manifold) The zero set *Z* of Ω on $[0,2\pi ]^3$ can be split into $Z = Z_+ \cup Z_-$, where

$$\begin{aligned}&Z_+ = Z \cap \{ 0 \le x + y - z \le 2\pi \} \\&Z_- = Z \cap \{ x+ y -z \le 0 \; \text{ or } \; x+ y - z \ge 2\pi \}. \end{aligned}$$

The set $Z_+$ consists of $(x,y,z) \in [0,2\pi ]^3$ such that $x=y \in \{0,2\pi \}$ or x=z or y=z.

As for $Z_-$, it can be described as follows: it consists of $(x,y,z) \in [0,2\pi ]^3$ such that

- either x<z and y=h(x,z)
- or x>z and y=h(x,z)+2π.

#### Proof

A basic inequality. We start by proving that

A.1 $$\begin{aligned} \left| \tan \left( \frac{z-x}{4} \right) \cos \left( \frac{x+z}{4} \right) \right| \le 1 \quad \text{ if } (x,z) \in [0,2\pi ]^2. \end{aligned}$$

This inequality is a consequence of

$$ \left| \cos \left( \frac{x+z}{4} \right) \right| \le \cos \left( \frac{x-z}{4} \right) \quad \text{ if } (x,z) \in [0,2\pi ]^2. $$

To check the latter inequality, we observe that, on the one hand, $0 \le \left| \frac{x-z}{4} \right| \le \frac{x+z}{4} \le \pi$, which implies that

A.2 $$\begin{aligned} \cos \left( \frac{x+z}{4} \right) \le \cos \left( \frac{x-z}{4} \right) . \end{aligned}$$

On the other hand, $0 \le \left| \frac{x-z}{4} \right| \le \frac{4 \pi - x - z}{4} \le \pi$, which implies that

A.3 $$\begin{aligned} \cos \left( \pi - \frac{x+z}{4} \right) = -\cos \left( \frac{x+z}{4} \right) \le \cos \left( \frac{x-z}{4} \right) . \end{aligned}$$

${{\mathrm{Case 1: 0}} \le x + y - z \le 2\pi .}$ Then Ω can be written as

$$ \Omega (x,y,z) = \sin \left( \frac{x}{2} \right) + \sin \left( \frac{y}{2} \right) - \sin \left( \frac{z}{2} \right) - \sin \left( \frac{x+y-z}{2} \right) , $$

which becomes after using the trigonometric sum-to-product formulas

$$ \Omega (x,y,z) = 4 \sin \left( \frac{x+y}{4} \right) \sin \left( \frac{x-z}{4} \right) \sin \left( \frac{y-z}{4} \right) . $$

The description of $Z_+$ follows immediately from this formula.

${{\mathrm{Case 2:}} x+ y -z \le 0 {\textrm{or}} x+ y - z \ge 2\pi }$. The formula for Ω is now

$$ \Omega (x,y,z) = \sin \left( \frac{x}{2} \right) + \sin \left( \frac{y}{2} \right) - \sin \left( \frac{z}{2} \right) + \sin \left( \frac{x+y-z}{2} \right) , $$

which reduces, after using the trigonometric sum-to-product formulas, to

A.4 $$\begin{aligned} \Omega (x,y,z) = 2 \sin \left( \frac{x-z}{4} \right) \cos \left( \frac{x+z}{4} \right) + 2 \cos \left( \frac{x-z}{4} \right) \sin \left( \frac{x + 2y-z}{4} \right) . \end{aligned}$$

${{\mathrm{Case 2.1:}} x+y-z \le 0.}$ Note that this implies that

A.5 $$\begin{aligned} -\frac{\pi }{2} \le \frac{x-z}{4} \le 0 \qquad \text{ and } \qquad -\frac{\pi }{2} \le \frac{x+2y-z}{4} \le \frac{\pi }{2}. \end{aligned}$$

Using the formula (A.4), (*x*, *y*, *z*) is a zero of Ω if and only if

A.6 $$\begin{aligned} \sin \left( \frac{x + 2y-z}{4} \right) = \tan \left( \frac{z-x}{4} \right) \cos \left( \frac{x+z}{4} \right) . \end{aligned}$$

With the help of (A.1) and (A.5), the sin function can be inverted to get

$$ \frac{x + 2y-z}{4} = \arcsin \left[ \tan \left( \frac{z-x}{4} \right) \cos \left( \frac{x+z}{4} \right) \right] , $$

which can also be written as y=h(x,z). This shows that any solution of Ω(x,y,z)=0 with x+y-z≤0 is of the form y=h(x,z).

Conversely, if x≤z, we want to check that there exists a solution (in *y*) of Ω(x,y,z)=0 with x+y-z≤0. Thus, *y* is restricted to belong to satisfy this inequality, and to belong to [0,2π], or in other words, $0 \le y \le z-x$. But Ω changes sign between the two endpoints: indeed, by (A.2), (A.3), and (A.5),

$$\begin{aligned}&\Omega (x,y=0,z) = 2 \sin \left( \frac{x-z}{4} \right) \left[ \cos \left( \frac{x+z}{4} \right) + \cos \left( \frac{x-z}{4} \right) \right] \le 0 \\&\Omega (x,y=z-x,z) = 2 \sin \left( \frac{x-z}{4} \right) \left[ \cos \left( \frac{x+z}{4} \right) - \cos \left( \frac{x-z}{4} \right) \right] \ge 0. \end{aligned}$$

Thus, $y \mapsto \Omega (x,y,z)$ has at least a zero, which was the desired statement.

${{\mathrm{Case 2.2:}} x+y-z \ge 2\pi .}$ This implies that

$$ 0 \le \frac{x-z}{4} \le \frac{\pi }{2} \qquad \text{ and } \qquad \frac{\pi }{2} \le \frac{x+2y-z}{4} \le \frac{3\pi }{2}. $$

As a consequence, inverting the sin function in (A.6) with the help of (A.1) gives

$$ \frac{x + 2y-z}{4} = \pi - \arcsin \left[ \tan \left( \frac{z-x}{4} \right) \cos \left( \frac{x+z}{4} \right) \right] , $$

which is equivalent to y=h(x,z)+2π.

Conversely, if x≥z, we need to check that there exists a solution (in *y*) of Ω(x,y,z)=0 with $x+y-z \ge 2\pi$. Besides this inequality, the variable *y* is constrained to belong to [0,2π]; in other words, *y* ranges in $[2\pi + z - x, 2\pi ]$. There remains to check that Ω changes sign between these two endpoints; this is the case since

$$\begin{aligned}&\Omega (x,y=2\pi ,z) = 2 \sin \left( \frac{x-z}{4} \right) \left[ \cos \left( \frac{x+z}{4} \right) - \cos \left( \frac{x-z}{4} \right) \right] \le 0 \\&\Omega (x,y=2\pi +z-x,z)= 2 \sin \left( \frac{x-z}{4} \right) \left[ \cos \left( \frac{x+z}{4} \right) + \cos \left( \frac{x-z}{4} \right) \right] \ge 0. \end{aligned}$$

□

#### Lemma 13

(Integration on the resonant manifold with $p_2$ as integration variable) For a test function φ,

$$ \int _{\mathbb {T}^2} \delta ( \Omega (x,y,z)) \varphi (x,y,z) \,dy \,dz = \int _0^{2\pi } \varphi (x,h(x,z),z) \frac{2 \, \textrm{d}z}{\sqrt{F_+(x,z)}}, $$

(considering φ as periodic in *y*, *z*) where

$$ F_+(x,z) = \left[ \cos \left( \frac{x}{2} \right) + \cos \left( \frac{z}{2} \right) \right] ^2 + 4 \sin \left( \frac{x}{2} \right) \sin \left( \frac{z}{2} \right) . $$

#### Proof

Since *h*(*x*, *z*) is the unique zero (in *y*) of Ω(x,y,z),

$$ \delta (\Omega (x,y,z)) \, \textrm{d}y = \frac{1}{|\partial _y \Omega (x,h(x,z),z)|} \delta (y - h(x,z)). $$

The derivative of Ω is

$$ \partial _y \Omega (x,y,z) = \frac{1}{2} \left[ \cos \left( \frac{y}{2} \right) + \cos \left( \frac{x+y-z}{2} \right) \right] = \cos \left( \frac{x+2y-z}{4} \right) \cos \left( \frac{x-z}{4} \right) . $$

Evaluating this function at y=h(x,z) and using the definition of *h*, we find that

$$\begin{aligned} | \partial _y \Omega (x,h(x,z),z) |&= \cos \left( \frac{x-z}{4} \right) \sqrt{ 1 - \sin \left( \frac{x+2h-z}{4} \right) ^2 } \\&= \cos \left( \frac{x-z}{4} \right) \sqrt{ 1 - \tan \left( \frac{x-z}{4} \right) ^2 \cos \left( \frac{x+z}{4} \right) ^2 } \\&= \sqrt{ \cos \left( \frac{x-z}{4} \right) ^2 - \sin \left( \frac{x-z}{4} \right) ^2 \cos \left( \frac{x+z}{4} \right) ^2 }. \end{aligned}$$

If remains to check that

$$ \cos \left( \frac{x-z}{4} \right) ^2 - \sin \left( \frac{x-z}{4} \right) ^2 \cos \left( \frac{x+z}{4} \right) ^2 = \frac{1}{4} \left[ \cos \left( \frac{x}{2} \right) + \cos \left( \frac{z}{2} \right) \right] ^2 + \sin \left( \frac{x}{2} \right) \sin \left( \frac{z}{2} \right) . $$

This can be seen as follows: starting from the expression on the left-hand side, use the formulas $2 \cos ^2 x = 1 + \cos (2x)$ and $2 \sin ^2 x = 1 - \cos (2x)$ and then expand the resulting expression using that $\cos (a+b) = \cos a \cos b - \sin a \sin b$. □

#### Lemma 14

(Integration on the resonant manifold with $p_1$ as integration variable) For a test function φ, periodic in $\mathbb {T}^2$,

$$ \int _{\mathbb {T}^2} \delta ( \Omega (x,y,z)) \varphi (x,y) \, \, \textrm{d}y \, \, \textrm{d}z = 2 \int \varphi (x,y) \frac{\mathbb {1}_{F_-(x,y)>0}}{\sqrt{F_-(x,y)}} \, \textrm{d}y, $$

where

$$ F_-(x,z) = \left[ \cos \left( \frac{x}{2} \right) + \cos \left( \frac{z}{2} \right) \right] ^2 - 4 \sin \left( \frac{x}{2} \right) \sin \left( \frac{z}{2} \right) . $$

#### Proof

Given Lemma 13, it suffices to show that

$$ \int _{[0,2\pi ]} \varphi (x,y) \frac{ \mathbb {1}_{F_-(x,y)>0}}{\sqrt{F_-(x,y)}} \, \textrm{d}y = \int _{[0,2\pi ]} \varphi (x,h(x,z)) \frac{ \, \textrm{d}z }{\sqrt{F_+(x,z)}} $$

if one of these integrals is absolutely convergent. We basically want to perform a change of variables for fixed *x*, to y=h(x,z). We first notice that from Lemma 13 and due to the relation

$$\begin{aligned} \partial _2 \Omega \vert _{Z_-}(x, h(x,z),z) \partial _z h(x,z) +\partial _z \Omega \vert _{Z_-}(x, h(x,z),z) =0, \end{aligned}$$

we get

$$\begin{aligned} \vert \partial _z \Omega \vert _{Z_-}(x, h(x,z),z) \vert = \vert \partial _z h(x,z) \vert \frac{1}{2} \sqrt{F_+(x,z)}. \end{aligned}$$

We will now show that for *z* so that h(x,·) is locally invertible, it holds that

$$\begin{aligned} \vert \partial _z \Omega \vert _{Z_-}(x, y,z) \vert = \frac{1}{2}\sqrt{F_-(x,y)}, \end{aligned}$$

which finishes the claim. We are using the following ingredients:

- the fact that in order to construct all possible local inverse functions $\tilde{h}(y,x)$, of h(x,·), it is enough to find all *z*’s so that (for fixed *x*, *y*), it holds $(x,y,z) \in Z_-$ and $\Omega \vert _{Z_-} =0$.
- We then use the formula for $\Omega \vert _{Z_-}$:
  $$\Omega \vert _{Z_-} (x,y,z) = 2 \left( \sin \left( \frac{x+y}{4}\right) \cos \left( \frac{x-y}{4}\right) + \cos \left( \frac{x+y}{4}\right) \sin \left( \frac{x+y-2z}{4}\right) \right) , $$
  which yields after manipulations of trigonometric functions that $F_-(x,y)\ge 0$ is a necessary condition in order to have $\Omega \vert _{Z_-}=0$. Indeed one observes that if $\Omega \vert _{Z_-}=0$, then
  A.7 $$\begin{aligned} \sin \left( \frac{x+y-2z}{4} \right) = -\tan \left( \frac{x+y}{4} \right) \cos \left( \frac{x-y}{4} \right) , \end{aligned}$$
  which is valid (for $x,y,z \in \mathbb {R}$) only if $\frac{1}{4}F_-(x,y)\ge 0$, since $F_-(x,y)\ge 0$ is equivalent to $1 \ge \cos ^2 \left( \frac{x-y}{4}\right) \tan ^2 \left( \frac{x+y}{4} \right)$,.
- Whenever $F_- >0$, there are exactly two solutions in $Z_-$ to the energy problem, explicitly given by
  $$\begin{aligned} z_\sigma= &   \tilde{h}_\sigma (y,x) = \frac{x+y}{2} + 2 \sigma \arcsin \left( \tan \left( \frac{x+y}{4} \right) \cos \left( \frac{x-y}{4} \right) \right) \\  &   + 2\pi \mathbb {1}_{\sigma =-1} (-1)^{\mathbb {1}_{x+y>2\pi }} \,\ \sigma \in \{\pm 1\}. \end{aligned}$$
  These also satisfy (A.7).

Now we may compute the Jacobian of the change of variables in the class of these *z* where explicit calculations yield

A.8 $$\begin{aligned} \begin{aligned} \left| \partial _z \Omega _- \right| = \left| \cos \left( \frac{x+y}{4}\right) \right| \left| \cos \left( \frac{x+y-2z}{4}\right) \right|&= \left| \cos \left( \frac{x+y}{4}\right) \right| \left| \sqrt{1-\sin ^2\left( \frac{x+y-2z}{4} \right) } \right| \\&= \frac{1}{2} \sqrt{F_-(x,y)}. \end{aligned} \end{aligned}$$

Also note that for fixed *x*, there are at most two *y*’s that satisfy $F_-=0$, so the calculation in (A.8) holds up to a finite number of *y*’s. □

#### Lemma 15

(Vanishing rate of $$F_-$$) For fixed $$x \in (0,2\pi )$$, the function $$y \mapsto F_-(x,y)$$ has two zeros, $y^{\prime }<y^{\prime \prime }$, such that

$$ 0< y'< 2\pi - x< y'' < 2\pi . $$

The function is negative between these zeros, and furthermore,

A.9 $$\begin{aligned} {\left\{ \begin{array}{ll} & F_-(x,y) \gtrsim (y'-y)\operatorname {sin} \left( \frac{x}{2}\right) ,\ \text { on } [0, y')\\ & F_-(x,y) \gtrsim (y-y'') \operatorname {sin} \left( \frac{x}{2}\right) ,\ \text { on } (y'', 2\pi ] \end{array}\right. } \end{aligned}$$

#### Proof

By symmetry of $F_-$ (namely, the identity $F_-(2\pi -x,2\pi -y) = F_-(x,y)$), it suffices to consider the case $0 \le x \le \pi$. We will denote that

$$ c = \cos \left( \frac{x}{2} \right) \ge 0, \qquad s = \sin \left( \frac{x}{2} \right) \ge 0. $$

${{\textrm{The derivatives of}} F_-.}$ Differentiating in *y* gives

$$ F_2(x,y) = [\partial _y F_{{-}}](x,y) = - \sin \left( \frac{y}{2} \right) \left( \cos \left( \frac{x}{2} \right) + \cos \left( \frac{y}{2} \right) \right) - 2 \sin \left( \frac{x}{2} \right) \cos \left( \frac{y}{2} \right) . $$

At this point, it is convenient to switch to the variable $u = \cos \left( \frac{y}{2} \right)$. Abusing notations, we write that

$$ F_2(x,u) = F_2(x,y(u)) = - \sqrt{1 - u^2}(c+u) - 2 u \sqrt{1-c^2}. $$

Taking further derivatives in *u*,

$$\begin{aligned}&\partial _u F_2(x,u) = \frac{2u^2+uc-1}{\sqrt{1-u^2}} - 2 \sqrt{1-c^2} \\&\partial _u^2 F_2(x,u) = \frac{P(u)}{(1-u^2)^{\frac{3}{2}}}, \qquad P(u) = -2u^3+3u+c \\&P'(u) = -6u^2 +3. \end{aligned}$$

$${{\textrm{Sign of}} \partial _u^2 F_2(x,\cdot ).}$$ Since $P^{\prime }$ has roots at $$- 2^{-\frac{1}{2}}$$ and $$2^{-\frac{1}{2}}$$, the former is a local minimum of *P* and the latter a local maximum. Furthermore, P(-1)=c-1<0, P(0)=c>0 and $$P(1) = c+1 {>}0$$. As a consequence, *P* has a unique root $$u_0<0$$ in [-1,1], and P>0 if and only if $$u>u_0$$; hence the same holds true for $$\partial _u^2 F_2(x,\cdot )$$.

${{\textrm{Sign of}} \partial _u F_2(x,\cdot ).}$ By the previous paragraph, $\partial _u F_2(x,\cdot )$ is decreasing if $u<u_0$, and increasing if $u>u_0$. Furthermore, $\partial _u F_2(-1) = {{+}}\infty$, $\partial _u F_2(0) = -1-2\,s<0$ and $\partial _u F_2(1) = + \infty$. Therefore, $\partial _u F_2(x,\cdot )$ has two zeros, $u_-<0$ and $u_+>0$, and $\partial _u F_2(x,\cdot )>0$ if and only if $u<u_-$ or $u>u_+$.

${{\textrm{Sign of}} F_2(x,\cdot ).}$ Coming back to the *y* variable, we learn from the previous paragraph that $y\mapsto F_2(x,y)$ is increasing on $[y_-,y_+]$, with $y_-<\pi <y_+$, and decreasing otherwise ($y_{\pm }$ corresponds to $u_{\mp }$). Next, we note that $F_2(x,0) = -2\,s <0$, $F_2(x,\pi ) = -c <0$ and $F_2(x,2\pi ) = 2 s >0$. Therefore, there exists $y_0>\pi$ such that $F_2(x,y)>0$ if and only if $y>y_0$.

$${{\textrm{Sign of}} F_-(x,\cdot ).}$$ The previous paragraph tells us that $$F_-(x,\cdot )$$ is decreasing on $$[0,y_0]$$ and increasing on $$[y_0,2\pi ]$$, with $$y_0>\pi $$. Since $$F_-(x,0)=(1+c)^2>0$$, $$F_-(x,2\pi -x) = - 4\,s^2<0$$ and $$F_-(x,2\pi ) = (c-1)^2 >0$$, there exists $y^{\prime }$ and $y^{\prime \prime }$ such that $$F_-(x,\cdot ) <0$$ if and only if $y\in (y^{\prime },y^{\prime \prime })$ and furthermore $$0<y'<2\pi -x<y''<2\pi $$ and $$y'<y_0<y''$$.

Reformulation of the problem. Writing that

$$ F_-(x,y) = {\left\{ \begin{array}{ll} - \int _y^{y'} F_2(x,z) \, dz &  \text{ if } y<y'\\ \int _{y''}^y F_2(x,z) \,dz &  \text{ if } y>y'', \end{array}\right. } $$

we see that it suffices to show that

$$ {\left\{ \begin{array}{ll} F_2(x,y) \lesssim -s &  \text{ if } y<y'\\ F_2(x,y) \gtrsim s &  \text{ if } y>y''. \end{array}\right. } $$

Since $F_2$ is increasing on $[y_-,y_+]$ and decreasing on $[y_+,2\pi ]$ and since $y'<y_0<y''$,

$$ \text{ on } [y'',2\pi ], \qquad F_2(x,y) \ge \min (F_2(x,y''),F_2(x,2\pi )) = \min (F_2(x,y''),2\,s). $$

Similarly,

$$ \text{ on } [0,y'], \qquad F_2(x,y) \le \max (F_2(x,y'),F_2(x,0)) = \max (F_2(x,y'),-2\,s). $$

By the two previous assertions, it suffices to show that

$$ F_2(x,y') \lesssim -s \quad \text{ and } \quad F_2(x,y'') \gtrsim s. $$

End of the proof. We now refer to the argument in [11], equations (4.12) to (4.17), a clever sequence of inequalities which we were not able to simplify. □

### A.2: Asymptotics of the collision frequency

#### Lemma 16

(Asymptotics of the collision frequency for zero mass) If γ=0, *a*(*p*) is a nonnegative continuous functions on $\mathbb {T}$ vanishing only at 0. Furthermore,

$$ a(p) \sim \sin \left( \frac{p}{2} \right) ^{5/3} \qquad \text{ if } p \in [0,2\pi ]. $$

#### Proof

It follows from the Definition (4.2), along with Lemmas (12) and (13), that

$$ a(x) = \omega (x)^2 \int _0^{2\pi } \frac{2 \, \textrm{d}z}{\sqrt{ \left( \cos \left( \frac{x}{2} \right) + \cos \left( \frac{z}{2} \right) \right) ^2 + 4 \sin \left( \frac{x}{2} \right) \sin \left( \frac{z}{2} \right) }}. $$

First observe that a(x)=a(2π-x), hence it suffices to consider the case $x \in [0,\pi ]$. Second, as long as *x* does not approach 0, it is clear that a(x)∼1. Hence, it suffices to compute the equivalent of the integral as x→0. The contribution of this integral for $z < \frac{3\pi }{2}$ is bounded; indeed, the denominator is bounded from below, since

$$ \cos \left( \frac{x}{2} \right) + \cos \left( \frac{z}{2} \right)> \cos \left( \frac{\pi }{8} \right) - \cos \left( \frac{\pi }{4} \right) > 0 \quad \text{ if } x \in (0,\frac{\pi }{4}) \;\; \text{ and } \;\; z \in (0,\frac{3\pi }{2}). $$

Therefore, we restrict the integration variable to $$(\frac{3\pi }{2},2\pi )$$ and then change variable to $z^{\prime }=2\pi -z$ to obtain the expresssion

$$ \int _0^{\frac{\pi }{2} } \frac{2 \, \textrm{d}z'}{\sqrt{ \left( \cos \left( \frac{x}{2} \right) - \cos \left( \frac{z'}{2} \right) \right) ^2 + 4 \sin \left( \frac{x}{2} \right) \sin \left( \frac{z'}{2} \right) }}. $$

Denoting $\epsilon = \sin \left( \frac{x}{2} \right)$ and $\sin \left( \frac{z'}{2} \right) = s$, the expression in the square root in the denominator can now be written as

$$\begin{aligned}&\left( \cos \left( \frac{x}{2} \right) - \cos \left( \frac{z'}{2} \right) \right) ^2 + 4 \sin \left( \frac{x}{2} \right) \sin \left( \frac{z'}{2} \right) \\&\qquad \qquad = \left( \frac{\sin \left( \frac{x}{2} \right) ^2 - \sin \left( \frac{z'}{2} \right) ^2}{\sqrt{1 - \sin \left( \frac{x}{2} \right) ^2} + \sqrt{1 - \sin \left( \frac{z'}{2} \right) ^2}} \right) ^2 + 4 \sin \left( \frac{x}{2} \right) \sin \left( \frac{z'}{2} \right) \\&\qquad \qquad = \left( \frac{\epsilon ^2 - s^2 }{\sqrt{1-\epsilon ^2} + \sqrt{1 -s^2 }} \right) ^2+ 4 \epsilon s. \end{aligned}$$

The integral becomes

$$\begin{aligned}&\int _0^{\sqrt{2}/2} \left[ \left( \frac{\epsilon ^2 - s^2 }{\sqrt{1-\epsilon ^2} + \sqrt{1 -s^2 }} \right) ^2+ 4 \epsilon s \right] ^{-\frac{1}{2}} \frac{2 \, \textrm{d}s}{\sqrt{1 - s^2}} \\&\qquad \qquad \qquad \qquad \sim \int _0^{\sqrt{2}/2} \left[ \left( \epsilon ^2 - s^2 \right) ^2+ 4 \epsilon s \right] ^{-\frac{1}{2}} \, \textrm{d}s \sim \epsilon ^{-\frac{1}{3}}. \end{aligned}$$

Here, we used that

$$ \left( \epsilon ^2 - s^2 \right) ^2+ 4 \epsilon s \sim {\left\{ \begin{array}{ll} \epsilon ^4 &  \text{ if } s< \epsilon ^3\\ \epsilon s &  \text{ if } \epsilon ^3< s < \epsilon ^{\frac{1}{3}}\\ s^4 &  \text{ if } s> \epsilon ^{\frac{1}{3}}. \end{array}\right. } $$

□

#### Lemma 17

For any $$x,z \in [0,2\pi ]$$,

$$ \left| \sin \left( \frac{\underline{h}(x,z)}{2} \right) \sin \left( \frac{{h}(x,z)}{2} \right) \right| = \tan ^2\left( \frac{z-x}{4} \right) \sin \left( \frac{z}{2} \right) \sin \left( \frac{x}{2} \right) . $$

#### Proof

Denoting that

$$ g(x,z) = \arcsin \left( \tan \left( \frac{|z-x|}{4} \right) \cos \left( \frac{z+x}{4} \right) \right) , $$

we can write that

$$\begin{aligned} \left| \sin \left( \frac{\underline{h}(x,z)}{2} \right) \right|&= \left| \sin \left( g(x,z) - \frac{z-x}{4} \right) \right| = \left| \sin \left( \frac{z-x}{4} \right) \right| \left| \sigma \cos \left( \frac{z+x}{4} \right) - \cos (g(x,z)) \right| \\ \left| \sin \left( \frac{{h}(x,z)}{2} \right) \right|&= \left| \sin \left( g(x,z) + \frac{z-x}{4} \right) \right| = \left| \sin \left( \frac{z-x}{4} \right) \right| \left| \sigma \cos \left( \frac{z+x}{4} \right) + \cos (g(x,z)) \right| , \end{aligned}$$

where $$\sigma = {{\,\textrm{sgn}\,}}(z-x)$$.

As a result,

$$\begin{aligned}&\left| \sin \left( \frac{\underline{h}(x,z)}{2} \right) \sin \left( \frac{{h}(x,z)}{2} \right) \right| = \sin ^2 \left( \frac{z-x}{4} \right) \left| \cos ^2 \left( \frac{z+x}{4} \right) - \cos ^2 (g(x,z)) \right| \\&\qquad = \sin ^2 \left( \frac{z-x}{4} \right) \left[ \cos ^2 \left( \frac{z+x}{4} \right) - 1 + \tan ^2\left( \frac{z-x}{4} \right) \cos ^2 \left( \frac{z+x}{4} \right) \right] \\&\qquad = \sin ^2 \left( \frac{z-x}{4} \right) \frac{\cos ^2 \left( \frac{z+x}{4} \right) - \cos ^2 \left( \frac{z-x}{4} \right) }{\cos ^2 \left( \frac{z-x}{4} \right) } = \tan ^2\left( \frac{z-x}{4} \right) \sin \left( \frac{z}{2} \right) \sin \left( \frac{x}{2} \right) . \end{aligned}$$

□

#### Lemma 18

(Asymptotics of the collision frequency for non-zero mass) If γ>0, *a*(*p*) is a nonnegative continuous functions on $\mathbb {T}$ vanishing only at 0. Furthermore,

$$ a(p) \sim \sin \left( \frac{p}{2} \right) ^{5/3} \qquad \text{ if } p \in [0,2\pi ]. $$

#### Proof

It follows from the Definition (4.2), Lemma (12) and (13) that

A.10 $$\begin{aligned} a(x) \sim \sin \left( \frac{x}{2} \right) \int _0^{2\pi } \frac{ \sin \left( \frac{z}{2} \right) \left| \sin \left( \frac{h(x,z)}{2} \right) \sin \left( \frac{\underline{h}(x,z)}{2} \right) \right| }{\sqrt{ \left( \cos \left( \frac{x}{2} \right) + \cos \left( \frac{z}{2} \right) \right) ^2 + 4 \sin \left( \frac{x}{2} \right) \sin \left( \frac{z}{2} \right) }} \, \textrm{d}z \end{aligned}$$

As in the previous lemma, it suffices to consider the case x→0. By Lemma 17,

A.11 $$\begin{aligned} \left| \sin \left( \frac{z}{2} \right) \sin \left( \frac{\underline{h}(x,z)}{2} \right) \sin \left( \frac{{h}(x,z)}{2} \right) \right| = \tan ^2 \left( \frac{z-x}{4} \right) \sin ^2 \left( \frac{z}{2} \right) \sin \left( \frac{x}{2} \right) . \end{aligned}$$

Setting $z^{\prime }=2\pi -z$, this becomes

A.12 $$\begin{aligned} \begin{aligned}&\left| \sin \left( \frac{z}{2} \right) \sin \left( \frac{\underline{h}(x,z)}{2} \right) \sin \left( \frac{{h}(x,z)}{2} \right) \right| = \cot ^2\left( \frac{z'+x}{4}\right) \sin ^2 \left( \frac{z'}{2} \right) \sin \left( \frac{x}{2} \right) \\&\qquad \qquad = \frac{ \left[ 1+ \cos \left( \frac{z'}{2} \right) \cos \left( \frac{x}{2} \right) - \sin \left( \frac{z'}{2} \right) \sin \left( \frac{x}{2} \right) \right] \sin ^2 \left( \frac{z'}{2} \right) \sin \left( \frac{x}{2} \right) }{1 - \cos \left( \frac{x}{2} \right) \cos \left( \frac{z'}{2} \right) + \sin \left( \frac{x}{2} \right) \sin \left( \frac{z'}{2} \right) }. \end{aligned} \end{aligned}$$

$\underline{{\textrm{The case}} 0< z < 2\pi -c_0}$ (where $c_0 >0$). In that case, $\cos \left( \frac{z-x}{4} \right) \gtrsim 1$, and thus

$$ \left| \sin \left( \frac{z}{2} \right) \sin \left( \frac{\underline{h}(x,z)}{2} \right) \sin \left( \frac{{h}(x,z)}{2} \right) \right| \lesssim \sin \left( \frac{x}{2} \right) . $$

By (A.11) and the argument in Lemma 16, we see that the integrand in (A.10) is *O*(*x*), so that the contribution to *a*(*x*) is $O(x^2)$.

$\underline{{\textrm{The case}} z > 2\pi - c_0.}$ Letting $\epsilon = \sin \left( \frac{x}{2} \right)$ and $s = \sin \left( \frac{z'}{2} \right)$, formula (A.12) becomes

$$\begin{aligned} \left| \sin \left( \frac{z}{2} \right) \sin \left( \frac{\underline{h}(x,z)}{2} \right) \sin \left( \frac{{h}(x,z)}{2} \right) \right|&= \frac{\epsilon s^2 \left( 1-\epsilon s + \sqrt{1-\epsilon ^2}\sqrt{1-s^2} \right) }{1 - \sqrt{1-\epsilon ^2} \sqrt{1- s^2} + \epsilon s } \\&\sim \frac{\epsilon s^2}{\epsilon ^2 + s^2} \sim {\left\{ \begin{array}{ll} \frac{s^2}{\epsilon }&  \text{ if } s< \epsilon \\ \epsilon &  \text{ if } s > \epsilon \end{array}\right. } \end{aligned}$$

Combining this equivalent with the proof of Lemma 16 gives the desired result. □

#### Lemma 19

For any $$x,z \in [0,2\pi ]$$,

$$ \left| \sin \left( \frac{z}{2} \right) \sin \left( \frac{h(x,z)}{2} \right) \sin \left( \frac{\underline{h}(x,z)}{2} \right) \right| \lesssim \sin \left( \frac{x}{2} \right) . $$

#### Proof

By Lemma 17,

$$ \left| \sin \left( \frac{z}{2} \right) \sin \left( \frac{h(x,z)}{2} \right) \sin \left( \frac{\underline{h}(x,z)}{2} \right) \right| = \tan ^2 \left( \frac{z-x}{4} \right) \sin ^2 \left( \frac{z}{2} \right) \sin \left( \frac{x}{2} \right) . $$

For (*x*, *z*) not close to (0,2π),

$$ \sin \left( \frac{z}{2} \right) \sin \left( \frac{h(x,z)}{2} \right) \sin \left( \frac{\underline{h}(x,z)}{2} \right) \lesssim \sin \left( \frac{x}{2} \right) . $$

For (*x*, *z*) close to (0,2π), we use the notation $\epsilon = \sin \left( \frac{x}{2} \right)$ and $s = \sin \left( \frac{z}{2} \right)$ to get, as in the proof of Lemma 18,

$$ \sin \left( \frac{z}{2} \right) \sin \left( \frac{h(x,z)}{2} \right) \sin \left( \frac{\underline{h}(x,z)}{2} \right) \sim \frac{\epsilon s^2}{\epsilon ^2 + s^2} \lesssim \epsilon = \sin \left( \frac{x}{2}\right) . $$

□
